# The existence of a nonclassical TCA cycle in the nucleus that wires the metabolic-epigenetic circuitry

**DOI:** 10.1038/s41392-021-00774-2

**Published:** 2021-11-03

**Authors:** Xujun Liu, Wenzhe Si, Lin He, Jianguo Yang, Yani Peng, Jie Ren, Xiaoping Liu, Tong Jin, Huajing Yu, Zihan Zhang, Xiao Cheng, Wenting Zhang, Lu Xia, Yunchao Huang, Yue Wang, Shumeng Liu, Lin Shan, Yu Zhang, Xiaohan Yang, Haixia Li, Jing Liang, Luyang Sun, Yongfeng Shang

**Affiliations:** 1 Key Laboratory of Carcinogenesis and Translational Research (Ministry of Education), Department of Biochemistry and Molecular Biology, Department of Laboratory Medicine, Peking University Third Hospital, School of Basic Medical Sciences, Peking University Health Science Center, 100191 Beijing, China; 2 Department of Clinical Laboratory, Peking University First Hospital, 100034 Beijing, China; 3 Department of Biochemistry and Molecular Biology, School of Basic Medical Sciences, Hangzhou Normal University, 311121 Hangzhou, China; 4 Department of Biochemistry and Molecular Biology, School of Basic Medical Sciences, Capital Medical University, 100069 Beijing, China; 5 Department of Integration of Chinese and Western Medicine, School of Basic Medical Sciences, Peking University Health Science Center, 100191 Beijing, China

**Keywords:** Biochemistry, Epigenetics

## Abstract

The scope and variety of the metabolic intermediates from the mitochondrial tricarboxylic acid (TCA) cycle that are engaged in epigenetic regulation of the chromatin function in the nucleus raise an outstanding question about how timely and precise supply/consumption of these metabolites is achieved in the nucleus. We report here the identification of a nonclassical TCA cycle in the nucleus (nTCA cycle). We found that all the TCA cycle-associated enzymes including citrate synthase (CS), aconitase 2 (ACO2), isocitrate dehydrogenase 3 (IDH3), oxoglutarate dehydrogenase (OGDH), succinyl-CoA synthetase (SCS), fumarate hydratase (FH), and malate dehydrogenase 2 (MDH2), except for succinate dehydrogenase (SDH), a component of electron transport chain for generating ATP, exist in the nucleus. We showed that these nuclear enzymes catalyze an incomplete TCA cycle similar to that found in cyanobacteria. We propose that the nTCA cycle is implemented mainly to generate/consume metabolic intermediates, not for energy production. We demonstrated that the nTCA cycle is intrinsically linked to chromatin dynamics and transcription regulation. Together, our study uncovers the existence of a nonclassical TCA cycle in the nucleus that links the metabolic pathway to epigenetic regulation.

## Introduction

The tricarboxylic acid (TCA) cycle, discovered by Hans Krebs in 1937 hence also called Krebs cycle or citric acid cycle, is a central hub for metabolism and energy production. Implemented with a series of biochemical reactions in mitochondrial matrix, the TCA cycle allows aerobic organisms to oxidize carbohydrates, fatty acids, and amino acids to provide energy, macromolecules, and redox balance to the cell.^1,2^ As the TCA cycle is of paramount importance to cell homeostasis, dysfunction of the TCA cycle has been linked to pathological conditions ranging from diabetes, neurodegeneration to cancer.^3–7^

Cell fate determination and tissue specification in eukaryotes are governed by spatiotemporal control of gene expression patterns in which exquisite and precise epigenetic mechanisms are implemented to ensure that the right genes are expressed at a right site in a right time.^8^ Along with DNA 5-cytosine methylation/demethylation, post-translational modifications of histone species including phosphorylation, acetylation, methylation, succinylation, ubiquitination, poly-ADP-ribosylation represent integral components of the hierarchical regulatory mechanisms in shaping the epigenetic landscape that dictates the gene expression pattern.^9–11^ Remarkably, a great variety of the mitochondrial TCA cycle metabolites are actively involved in the dynamics of DNA/histone modifications thus epigenetic regulations in the nucleus. For instance, histone acetylation and succinylation involves acetyl-CoA and succinyl-CoA, respectively. The amine oxidase family of histone demethylases, exemplified by LSD1, need FAD for proton transfer in its demethylation reaction,^12^ and the other family of histone demethylases, the Jumonji proteins, need α-ketoglutarate (α-KG) in their demethylation reactions,^13^ in which α-KG is converted to succinate. The requirement for α-KG and the conversion of α-KG to succinate also occur in DNA demethylation process, in which the TET dioxygenases catalyze a series oxidation reactions to convert 5-methylcytosine (5mC) to 5-hydroxymethylcytosine (5hmC), 5-formylcytosine (5fC), and 5-carboxylcytosine (5caC).^14^ In fact, over 60 α-KG-dependent dioxygenases are found in mammalian cells, with the majority of them functioning in the nucleus.^15^ Moreover, NAD^+^, another proton acceptor in the TCA cycle, is required in the nucleus by sirtuins and poly (ADP-ribose) polymerase (PARP) to catalyze deacetylation and poly-ADP-ribosylation of histones as well as nonhistone proteins, respectively.

The scope and variety of the TCA cycle metabolites and cofactors engaged in epigenetic mechanisms raise a distinct question about where these molecules come from and where do they go once produced in the nucleus. A simple and straightforward answer to this question is that all these metabolites and cofactors come from the TCA cycle in mitochondria, but this requires two premises: (1) a direct communication/sensor system exists between mitochondria and the nucleus; and (2) active transporters for these molecules are available in the cell. Although there is no evidence to support the existence of first one system in cells, the diffusions of metabolic intermediates via transporters to the nucleus for epigenetic regulation will be energy-consuming and indirect. Considering the tightly controlled and highly dynamic nature of epigenetic regulation, the cell must evolve an equally tightly regulated system for efficient and timely supply/removal of the TCA cycle intermediates in the nucleus. Making the issue more complex, the cell has to constantly respond to environmental changes such as stress, nutrient shortage, and hormone stimulation to adjust the cellular activities such as cell differentiation and cell division by tuning the chromatin activity in the nucleus. Such responses must be prompt and precise thus supplies from remote sources and via laborious delivery are probably not evolutionarily advantageous.

Despite the dogma that the TCA cycle enzymes reside and function in mitochondria, several recent studies independently reported the existence of the TCA cycle-associated enzymes also in the nucleus. Specifically, it was reported that pyruvate dehydrogenase (PDH) is also detected in the nucleus to feed local acetyl-CoA required for histone acetylation^16^; it was found that oxoglutarate dehydrogenase (OGDH) also exists in the nucleus, although nuclear OGDH was proposed to interact with GCN5 on chromatin to regulate histone succinylation;^17^ and, remarkably, several the TCA cycle-associated enzymes, including citrate synthase (CS), aconitase 2 (ACO2), isocitrate dehydrogenase 3 (IDH3), and PDH, were found in the nucleus in the 1-2 cell stages of pre-implanted mouse embryo to provide metabolic fuels to zygotic genome activation in the nucleus.^18^ It becomes imperative to have a systematic and comprehensive investigation about the existence and functionality of the TCA cycle enzymes in the nucleus, especially considering the importance and elaboration of the nucleus in the maintenance and translation of the genetic information, as described above.

In the current study, we tested the hypothesis that the cell nucleus possesses a self-sustained system to supply and consume the TCA cycle intermediates involved in the dynamics of DNA and histone modifications. We described the identification of a nonclassical and incomplete TCA cycle in the nucleus. We propose that the nuclear TCA cycle (nTCA cycle) is implemented for supply/consumption of the metabolites, not for energy production. We showed that the nTCA cycle is intrinsically linked to chromatin dynamics and transcription regulation.

## Results

### The catalytic enzymes of the TCA cycle are also found in the nucleus

**Fig. 1:**
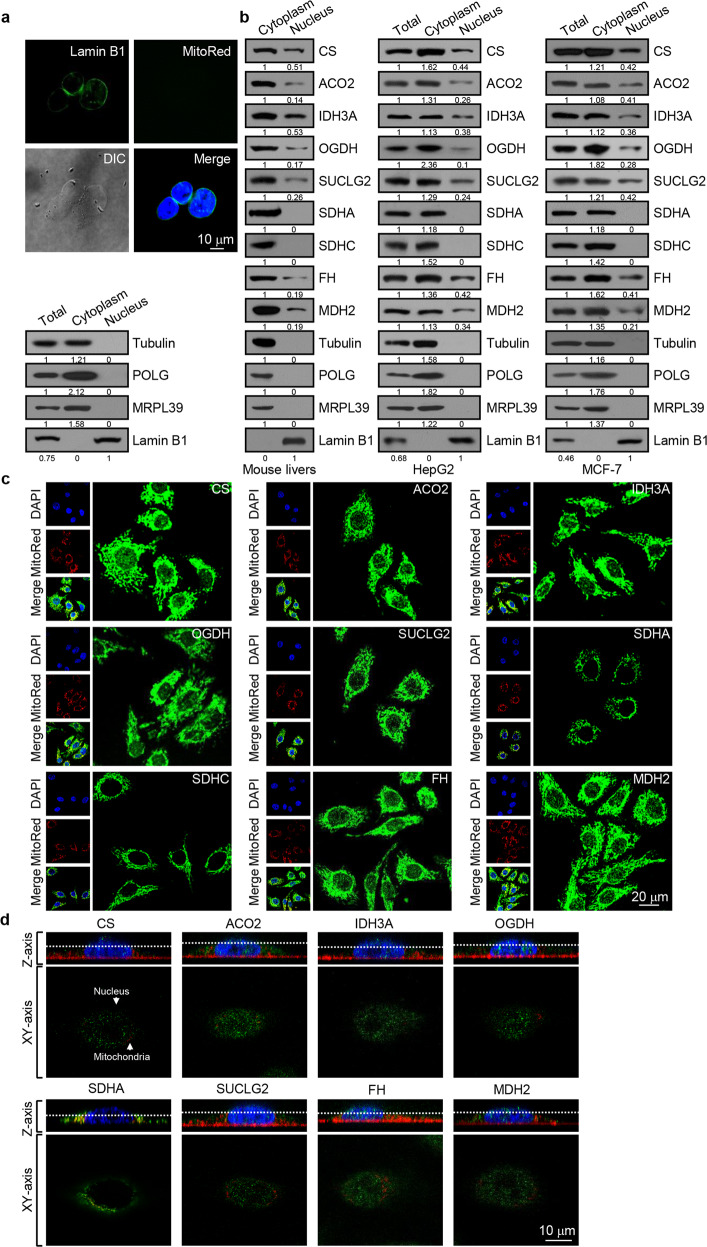
The enzymes of the TCA cycle are also found in the nucleus. **a** Nuclei were isolated from mouse livers using a nuclei-specific, high-sucrose gradient centrifugation protocol. The purity of the nuclei was verified by western blotting analysis of cytoplasmic and mitochondrial proteins. Mitochondria were stained with MitoTracker Red (red), and nuclei were stained with Lamin B1 (green) or DAPI (blue). Bar, 10 μm. **b** Western blotting analysis of the extracts from nuclei isolated from mouse livers, HepG2 cells, or MCF-7 cells using antibodies against the indicated proteins. **c** Subcellular localization of the TCA cycle-associated enzymes in HepG2 cells was analyzed by immunofluorescent staining (green). DAPI was used to stain the nucleus (blue), and MitoTracker Red staining was included to visualize the mitochondria (red). Bar, 20 μm. **d** HepG2 cells were immunostained with antibodies against the TCA cycle-associated enzymes (green) or MitoTracker Red (red) and scanned at a 0.5-μm increment along the *Z*-axis using confocal microscopy. DAPI staining was used to visualize the nucleus (blue). Bar, 10 μm

As stated above, several recent studies have reported the finding of the TCA cycle-associated enzymes in the nucleus.^16–18^ To have a comprehensive investigation on the scope and variety of the TCA cycle enzymes in the nucleus, we first utilized a nuclei-specific, high-sucrose gradient centrifugation protocol to isolate nuclei from mouse livers. The isolated nuclei were with intact membrane, judged on Lamin B1-staining pattern (Fig. 1a), free of mitochondria, manifested by the mitochondrial marker MitoTracker Red staining, and without contamination with cytoplasmic and mitochondrial proteins, evidenced by immunoblotting with antibodies against Tubulin, mitochondrial DNA polymerase POLG and mitochondrial ribosomal protein L39 (MRPL39), respectively (Fig. 1a). Consistently, immunofluorescent staining analysis showed that the fluorescent signal of neither POLG or MRPL39 was detected in the nucleus, confirming that the isolated nuclei were not contaminated by damaged mitochondria (Supplementary Fig. S1). Western blotting was then performed to detect the TCA cycle-associated enzymes using antibodies against CS, ACO2, IDH3A, OGDH, succinyl-CoA ligase GDP-forming beta subunit (SUCLG2), succinate dehydrogenase complex flavoprotein subunits A and C (SDHA and SDHC), fumarate hydratase (FH), and malate dehydrogenase 2 (MDH2) in the isolated mouse liver nuclei, as well as in nuclei isolated using the same protocol from human hepatocellular carcinoma cell line HepG2 and human mammary adenocarcinoma cell line MCF-7. Remarkably, all CS, ACO2, IDH3A, OGDH, SUCLG2, FH, and MDH2, except for SDHA and SDHC, were detected in the isolated mouse liver nuclei (Fig. 1b). Strikingly, a similar profile of the TCA cycle-associated enzymes was also detected in the isolated nuclei from HepG2 and MCF-7 cells (Fig. 1b).

To further support the above observations, immunofluorescent staining was performed in HepG2 cells using antibodies against CS, ACO2, IDH3A, OGDH, SUCLG2, SDHA, SDHC, FH, MDH2, and MitoTracker Red. The immunostaining of these enzymes and co-localization of these proteins with the mitochondrial signal were evident in mitochondria, as expected (Fig. 1c). Remarkably, immunofluorescent signal was also detected in HepG2 nuclei for CS, ACO2, IDH3A, OGDH, SUCLG2, FH, and MDH2, whereas immunofluorescent signal for SDHA and SDHC was not detected (Fig. 1c), consistent with the results from western blotting. Meanwhile, HepG2 cells were treated with siRNAs against CS, ACO2, IDH3A, OGDH, SUCLG2, SDHA, SDHC, FH or MDH2, and the immunofluorescent signals were decreased remarkably compared to the control, verifying specificity of used antibodies (Supplementary Fig. S2). In addition, we systematically scanned in the *Z*-axis at a 0.5 μm depth for each image and selected specific planes that cut through the nucleus from the Z stacked images, as shown in Fig. 1d. In *Z*-axis and *XY*-axis, CS was detected in mitochondria and co-localized with MitoTracker Red, whereas in the nucleus, the CS signal was still evident while MitoTracker Red signal was absent (Fig. 1d). Systematical imaging of ACO2, IDH3A, OGDH, SUCLG2, FH, and MDH2 showed a similar pattern, whereas SDHA was only seen in mitochondria (Fig. 1d). Importantly, the nuclear signals of CS, ACO2, IDH3A, OGDH, SUCLG2, FH, and MDH2 were co-localized with the signal of histone H3 (Fig. 1e), reinforcing the existence of these TCA cycle-associated enzymes in the nucleus. For all the confocal experiments, a secondary antibody-only control was included, which yielded no detectable signal (Supplementary Fig. S3). Collectively, these observations indicate that all the mitochondrial TCA cycle-associated enzymes CS, ACO2, IDH3A, OGDH, SUCLG2, FH, and MDH2, except for SDH, also exist in the nucleus.

### The nuclear TCA cycle-associated enzymes catalyze the TCA cycle-related biochemical reactions

**Fig. 2:**
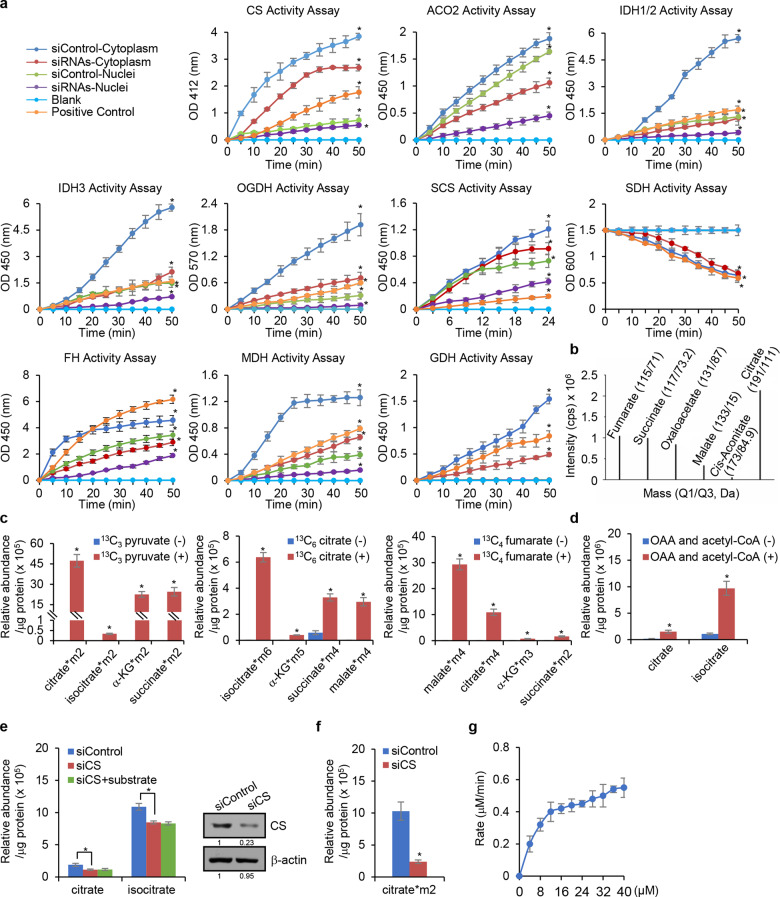
The nuclear TCA cycle-associated enzymes catalyze their corresponding biochemical reactions. **a** HepG2 cells treated with siRNA against control, CS, ACO2, IDH2, IDH3A, OGDH, SCS, SDHA, FH, MDH2 or GDH, and the nuclear and cytoplasmic extracts were prepared from HepG2 cells for enzymatic activity assays using the corresponding kit. Error bars represent mean ± SD for triplicate experiments (*p < 0.05). **b** The meta**b**olic intermediates related to the TCA cycle were detected in HepG2 nuclei by LC-MS. **c** HepG2 nu**c**lei were incubated with oxaloacetate and ^13^C_3_-pyruvate, ^13^C_6_-citrate, or ^13^C_4_-fumarate for detection of ^13^C-labeled-metabolic intermediates related to the TCA cycle by LC-MS. The data were normalized to protein concentration. Error bars represent mean ± SD for triplicate experiments (*p < 0.05). **d** HepG2 nuclei were incubated with or without oxaloacetate (OAA) and acetyl-CoA for detection of citrate and isocitrate by LC-MS. The data were normalized to protein concentration. Error bars represent mean ± SD for triplicate experiments (*p < 0.05). **e** Nuclei isolated from CS-deplet**e**d HepG2 cells were incubated with or without oxaloacetate and acetyl-CoA for detection of citrate and isocitrate by LC-MS. The data were normalized to protein concentration. Error bars represent mean ± SD for triplicate experiments (*p < 0.05). The efficiency of CS siRNA was determined by western blotting analysis. **f** Nuclei isolated from CS-depleted HepG2 cells were incubated with or without oxaloacetate and ^13^C_3_-pyruvate for detection of ^13^C-labeled-citrate by LC-MS. The data were normalized to protein concentration. Error bars represent mean ± SD for triplicate experiments. **g** Affinity-purified CS from HepG2 nuclei was incubated with 5 mM oxaloacetate and 0–40 μM acetyl-CoA, and the production of citrate was measured using the Citrate Colorimetric/Fluorometric Assay Kit. Error bars represent mean ± SD for triplicate experiments

To investigate the functional significance of the physical existence of the mitochondrial TCA cycle-associated enzymes in the nucleus, intact and pure nuclei were isolated from HepG2 cells using the nuclei-specific, high-sucrose gradient centrifugation protocol, and the enzyme activity for each of the detected TCA cycle enzymes in the nucleus, as well as in the cytoplasm was determined using a commercial enzyme activity assay kit. As expected, the enzymatic activity of CS, ACO2, IDH3, OGDH, SCS (SUCL), SDH, FH, and MDH2 was detected in the cytoplasm of HepG2 cells (Fig. 2a). Remarkably, the enzymatic activity of CS, ACO2, IDH3, OGDH, SCS, FH, and MDH2, except for SDH, was also detected in the nuclei of HepG2 cells (Fig. 2a). These results are consistent with the observations from western blotting and immunofluorescent staining, further substantiating the existence of the mitochondrial TCA cycle-associated enzymes in the nucleus. As a control, the mitochondrial matrix protein glutamate dehydrogenase (GDH) was not detected in the nuclei of HepG2 cells (Fig. 2a). Meanwhile, HepG2 cells were treated with siRNAs against control, CS, ACO2, IDH2, IDH3A, OGDH, SCS, SDHA, FH, MDH2 or GDH, and the enzyme activity for each enzyme in the nucleus, as well as in the cytoplasm was determined based on the colorimetry. The results showed that knockdown of these enzymes resulted in a decreased enzymatic activity in the cytoplasm and nuclei, except the decreased enzymatic activity for SDH and GDH was only detected in cytoplasm, verifying the specificity of enzyme activity assay (Fig. 2a).

The existence of the catalytically active TCA cycle-associated enzymes in the nucleus could mean that these enzymes act individually or independently. Alternatively, these enzymes and their catalyzed reactions constitute a biochemical system similar to the TCA cycle in mitochondria. To test this, we first isolated the nuclei from HepG2 cells and performed liquid chromatograph–mass spectrometry (LC-MS) to detect the mitochondrial TCA cycle-related metabolites in the nucleus. We found that indeed multiple the TCA cycle-related metabolic intermediates, including citrate, *cis*-aconitate, succinate, fumarate, malate, and oxaloacetate, were detected in isolated nuclei from HepG2 cells (Fig. 2b).

We then treated the nuclei isolated from HepG2 cells with oxaloacetate and isotope-labeled pyruvate (^13^C_3_-pyruvate). LC-MS analysis of the nuclear preparation detected ^13^C-labeled- mitochondrial TCA cycle intermediates including citrate, isocitrate, α-KG, and succinate (Fig. 2c), suggesting the existence of a systematic and the TCA cycle-like biochemical system in the nucleus. We also utilized isotope-labeled ^13^C_6_-citrate and ^13^C_4_-fumarate in the experiments, and LC-MS analysis also detected ^13^C-labeled-TCA intermediates in these conditions (Fig. 2c). Together, these observations indicate that a TCA cycle-like system is functioning in the nucleus.

To further support this, HepG2 nuclei were isolated and incubated with or without oxaloacetate and acetyl-CoA. LC-MS analysis showed that the levels of citrate and isocitrate increased in the nuclei added with oxaloacetate (OAA) and acetyl-CoA (Fig. 2d), indicating an active enzymatic reaction catalyzed by CS. In agreement, LC-MS analysis of the nuclei isolated from CS-depleted HepG2 cells showed that the levels of citrate and isocitrate decreased, compared to the nuclei isolated from control cells, and addition of oxaloacetate and acetyl-CoA did not result in increases in the levels of citrate and isocitrate (Fig. 2e). Likewise, LC-MS analysis showed that addition of oxaloacetate and ^13^C_3_-pyruvate to CS-depleted HepG2 nuclei was associated with a much lower level of ^13^C_2_-citrate, compared to the ^13^C_2_-citrate level in nuclei isolated from control siRNA-treated HepG2 (Fig. 2f). Collectively, the above results support the existence of a TCA cycle in the nucleus (nTCA cycle).

To further support the enzymatic reactions catalyzed by the nTCA cycle-associated enzymes, CS was affinity-purified from HepG2 nuclei and incubated with different concentrations of acetyl-CoA and a fixed concentration of oxaloacetate. Measurement of the reaction rate by monitoring the production of citrate indicated that citrate concentration increased linearly with acetyl-CoA concentration ranging from 0 to 14 μM (Fig. 2g). The increase of the citrate concentration became nonlinear when the concentration of acetyl-CoA reached to 16 μM and maximized (*V*_max_) when the concentration of acetyl-CoA reached to 36 μM (Fig. 2g). Lineweaver-Burk plotting calculated that the Michaelis constant (*K*_M_) of nuclear CS was 8.9 μM of acetyl-CoA, following the kinetics of a typical enzymatic reaction.

### The nTCA cycle is incomplete and different from the mitochondrial TCA cycle

The detection of CS, ACO2, IDH3A, OGDH, SUCLG2, FH, and MDH2, but not SDH, in the nucleus raises a question about how the TCA reactions cycle in the nucleus, as lack of SDH would result in succinate unable to convert to fumarate and render the TCA cycle incomplete. In this regard, it is interesting to note that cyanobacteria as well as some other prokaryotes also have an incomplete TCA cycle, although the incompleteness of the cycle is due to lack of OGDH.^19,20^ Apparently, the nTCA cycle is somewhat reminiscent of the cyanobacteria TCA cycle.

**Fig. 3:**
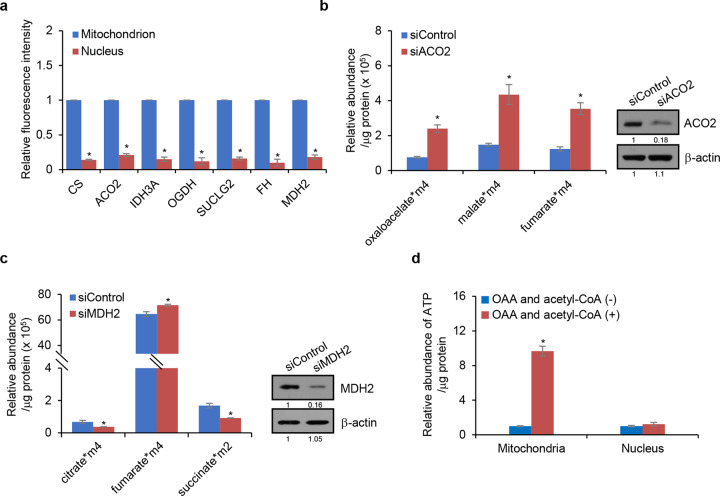
The nTCA cycle is incomplete and different from the mitochondrial TCA cycle. **a** HepG2 cells were immunostained with antibodies against CS, ACO2, IDH3A, OGDH, SUCLG2, FH, or MDH2. The mean immunofluorescent intensity of the indicated protein in mitochondria and nucleus was analyzed by Image J software. Error bars represent mean ± SD for triplicate experiments. **b** Nuclei isolated from ACO2-deficient HepG2 cells were incubated with ^13^C_6_-citrate and acetyl-CoA for detection of ^13^C-labeled metabolic intermediates related to the TCA cycle by LC-MS. The data were normalized to protein concentration. Error bars represent mean ± SD for triplicate experiments (*p < 0.05). Western blot analysis was utilized to determine the expression of indicated protein. **c** Nuclei isolated from MDH2-deficient HepG2 cells were incubated with ^13^C_4_-malate and acetyl-CoA for detection of ^13^C-labeled metabolic intermediates related to the TCA cycle by LC-MS. The data were normalized to protein concentration. Error bars represent mean ± SD for triplicate experiments (*p < 0.05). Western blot analysis was utilized to determine the expression of indicated protein. **d** Mitochondria and nuclei isolated from an equal number of HepG2 cells were incubated with oxaloacetate and acetyl-CoA for measurement of ATP production using the ATP Colorimetric/Fluorometric Assay Kit. Error bars represent mean ± SD for triplicate experiments (*p < 0.05)

To understand the nature of the nTCA cycle further, the nTCA cycle was compared with the classical mitochondrial TCA cycle. Immunofluorescent staining in HepG2 cells for CS showed that the signal of CS was much stronger in mitochondria than in the nucleus, suggesting that the abundance of CS is higher in mitochondria than in the nucleus (Fig. 3a). Similar experiments detecting ACO2, IDH3A, OGDH, SUCLG2, FH, and MDH2 showed that as a whole the abundance of these enzymes was higher in mitochondria than in the nucleus (Fig. 3a). These observations are not only interesting, but also informative: the TCA cycle enzymes are more enriched in mitochondria probably because this organelle is the main metabolic hub and the primary power house of the cell. In addition, the observation that the TCA cycle-associated enzymes are at a lower abundance in the nucleus might also explain why these enzymes as a whole have evaded detection before.

The central question is how the nTCA cycle skips the SDH-catalyzed reaction. In this regard, it is important to note that the majority of the enzymatic reactions in the classical TCA cycle are reversible, except for these that are catalyzed by CS, IDH3, and OGDH. Previous report has proven that ATP-citrate lyase (ACLY), the enzyme that converts citrate into acetyl-CoA, is present in the nuclei.^21^ In addition, as mentioned above, cyanobacteria also have an incomplete TCA cycle, and this organism does have MDH2, FH, SDH, and SUCL that are believed to catalyze the conversion of oxaloacetate to succinyl-CoA to produce malate, fumarate, and succinate in a reverse cycle.^19,20^ Based on the observation that the nTCA cycle lacks SDH and inspired by the incomplete TCA cycle in cyanobacteria, we propose that the nTCA cycle ends with succinate and the downstream metabolic intermediates of the SDH reaction, fumarate, malate, and oxaloacetate, are produced by reverse reactions similar to that in the cyanobacterial TCA cycle. In fact, it has been well-recognized even in mammalian cells that MDH2 could reverse oxaloacetate to malate and FH could reverse malate to fumarate.^22,23^ To test our hypothesis, we incubated ^13^C_6_-citrate and acetyl-CoA with nuclei isolated from ACO2-depleted HepG2 cells. LC-MS analysis indeed detected more ^13^C-labeled-oxaloacetate, malate, and fumarate from ACO2-depleted HepG2 cells (Fig. 3b). Analogously, incubation of ^13^C_4_-malate and acetyl-CoA with nuclei isolated from MDH2-depleted HepG2 cells resulted in detection of more ^13^C-labeled-fumarate but less succinate (Fig. 3c), further substantiating the observation that SDH is lacking in the nucleus. Together, these results indicate that although lack of SDH, the nTCA cycle is functional, and that the downstream metabolites of the SDH reaction, fumarate, malate and oxaloacetate, can be produced through a series of reverse reactions.

Next, we added the TCA cycle substrates oxaloacetate and acetyl-CoA to mitochondria and nuclei isolated from equal number of HepG2 cells. Detection of the production of ATP showed that the ATP level significantly increased in mitochondria upon addition of the substrates, whereas in the nucleus, addition of oxaloacetate and acetyl-CoA did not result in evident changes in the ATP level (Fig. 3d). This feature of the nTCA cycle is also reminiscent of that of the cyanobacterial TCA cycle, which is believed to primarily function to produce biosynthetic precursors for growth, not to generate energy.^19,20^ Collectively, the above results support the proposition that the nTCA cycle is a nonclassical, incomplete TCA cycle that mainly functions for generation of metabolic intermediates, but not for energy production.

### The nTCA cycle is functionally linked to epigenetic regulation and chromatin dynamics

**Fig. 4:**
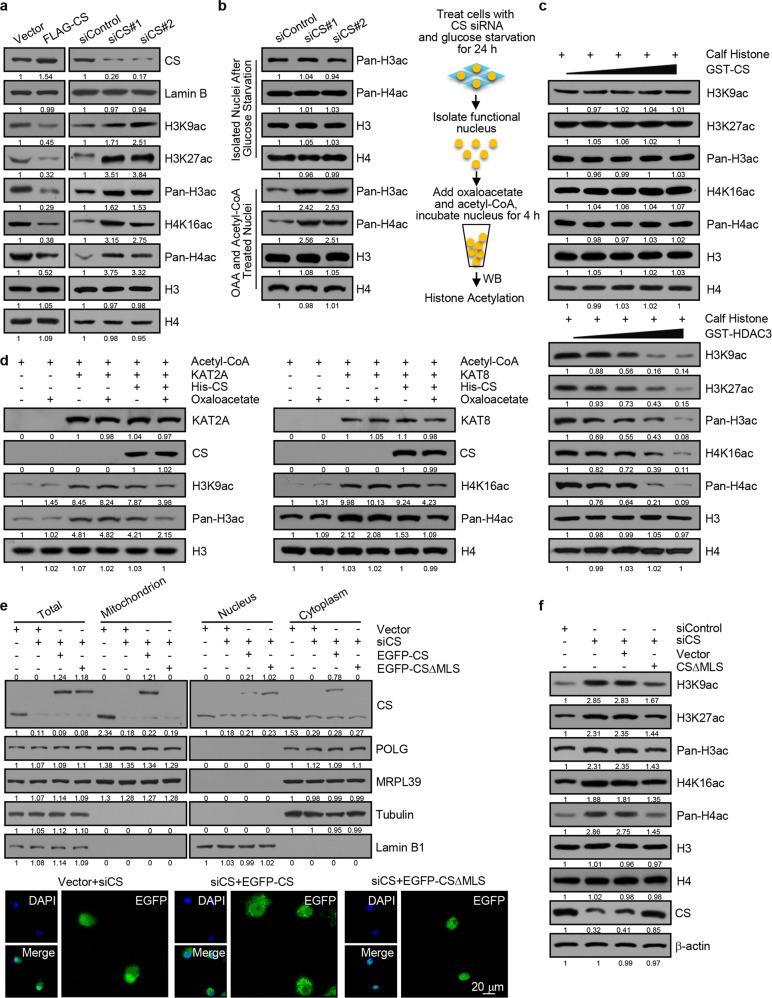
The nTCA cycle is functionally linked to epigenetic regulation and chromatin dynamics. **a** HepG2 cells were transfected with FLAG-CS or treated with CS siRNA, the nuclei were isolated and the level of the indicated histone marks or proteins was measured by western blotting. **b** CS-depleted HepG2 cells were deprived of glucose for 24 h. Nuclei were isolated and incubated with oxaloacetate and acetyl-CoA for western blotting analysis with antibodies against the indicated histone marks or proteins. The work flow for acetylation assays in isolated nuclei is shown. **c** In vitro deacetylation assays with bacterially expressed GST-fused proteins as indicated and calf thymus histones. The reaction was analyzed by western blotting with antibodies against the indicated histone marks or proteins. **d** KAT2A or KAT8 was incubated with calf thymus histones and acetyl-CoA in the presence or absence of oxaloacetate and with or without addition of affinity-purified CS from HepG2 nuclei. The reaction was analyzed by western blotting with antibodies against the indicated histone marks or proteins. **e** HepG2 cells treated with CS siRNA were transfected with EGFP-CS or EGFP-CS∆MLS for nuclei/cytoplasm/mitochondria isolation and western blotting analysis of CS expression. The subcellular localization of CS in these cells was analyzed by fluorescence microscopy. Bar, 20 μm. **f** HepG2 cells were transfected with CS siRNA and/or CS∆MLS for western blotting analysis with antibodies against the indicated histone marks or proteins

As stated earlier, a variety, if not all, of the mitochondrial TCA cycle metabolites have been found to actively participate in epigenetic regulation of chromatin activities. Given the existence of the nTCA cycle and its primary function in producing metabolic intermediates, it is reasonable to postulate that the nTCA cycle and the epigenetic regulation are functionally connected. To test this, CS, which catalyzes the reaction to produce citrate from oxaloacetate and acetyl-CoA, was overexpressed or knocked down in HepG2 cells, and the effect of CS on histone acetylation was analyzed by western blotting (Fig. 4a). Remarkably, overexpression of CS was associated with a decrease in the level of pan-H3 acetylation (pan-H3ac), as well as decreases in the levels of H3K9ac, H3K27ac, pan-H4ac, and H4K16ac, whereas knockdown of CS was accompanied by increases in the levels of these histone modifications (Fig. 4a). Moreover, the level of acetyl-CoA within the nuclear compartment upon CS overexpression or knockdown was detected using Acetyl-CoA Activity Assay Kit. The results showed that overexpression of CS led to the decreased level of acetyl-CoA, whereas depletion of CS resulted in an elevated level of acetyl-CoA in the nucleus (Supplementary Fig. S4), suggesting that nuclear CS impacts on histone acetylation.

It is believed that histone acetylation is largely influenced by metabolism of glucose, not free fatty acid.^21^ To further support the notion that nuclear CS influences histone acetylation, CS-depleted HepG2 cells were deprived of glucose for 24 h to synchronize histone acetylation. The nuclei were then isolated and exposed to oxaloacetate and acetyl-CoA. Western blotting showed that although the basal levels of pan-H3ac and pan-H4ac were similar in control nuclei versus CS-depleted nuclei, addition of oxaloacetate and acetyl-CoA led to increases in the levels of pan-H3ac and pan-H4ac only in CS-depleted nuclei (Fig. 4b). Moreover, in vitro deacetylation assays with GST-CS and calf thymus histones showed that CS had little effect on the levels of pan-H3ac, H3K9ac, H3K27ac, pan-H4ac, and H4K16ac, whereas incubating GST-HDAC3 with calf thymus histones led to reduced levels of pan-H3ac, H3K9ac, H3K27ac, pan-H4ac, and H4K16ac (Fig. 4c), arguing against a possibility that CS depletion-associated increases in pan-H3ac and pan-H4ac levels was a result of a diminished deacetylation activity of CS.

Next, we tested whether nuclear CS-influenced histone acetylation is histone acetyltransferase (HAT)-dependent. To this end, in vitro acetylation assays were performed by incubating acetyl-CoA and calf thymus histones with human recombinant KAT2A, a histone H3 HAT,^24^ in the presence or absence of oxaloacetate and CS that was affinity-purified from HepG2 nuclei. Western blotting analysis showed that the levels of KAT2A-catalyzed pan-H3ac as well as H3K9ac were significantly reduced when both oxaloacetate and CS were included in the reaction, whereas in the absence of oxaloacetate, CS had only marginal effect on the levels of pan-H3ac and H3K9ac (Fig. 4d), consistent with a role of CS to consume oxaloacetate and acetyl-CoA to produce citrate. Similar experiments were also performed with human recombinant KAT8, a histone H4 HAT.^25^ Western blotting revealed that likewise the levels of KAT8-catalyzed pan-H4ac as well as H4K16ac were significantly reduced, but only when both oxaloacetate and CS was present (Fig. 4d). These results indicate that nuclear CS could influence HAT-dependent histone acetylation by regulating the availability of local acetyl-CoA.

To further support nuclear CS-regulated histone modifications, we reconstituted nuclear expression of CS with the mitochondrial localization sequence-deleted CS (CS∆MLS). HepG2 cells treated with CS siRNAs were transfected with EGFP-tagged CS or CS∆MLS, and western blotting showed that the level of exogenously expressed CS or CS∆MLS was close to that of endogenous CS expression. The result showed that CS∆MLS was localized in nucleus, but not in mitochondria, whereas exogenous CS was mainly localized in mitochondrion, consistent with fluorescence microscopy (Fig. 4e). Western blotting analysis of acidly extracted histones showed that while depletion of endogenous CS was associated with an elevated level of histone acetylation, including pan-H3ac, H3K9ac, H3K27ac, pan-H4ac, and H4K16ac, reconstitution of nuclear expression of CS offset CS depletion-associated increases in histone acetylation (Fig. 4f).

**Fig. 5:**
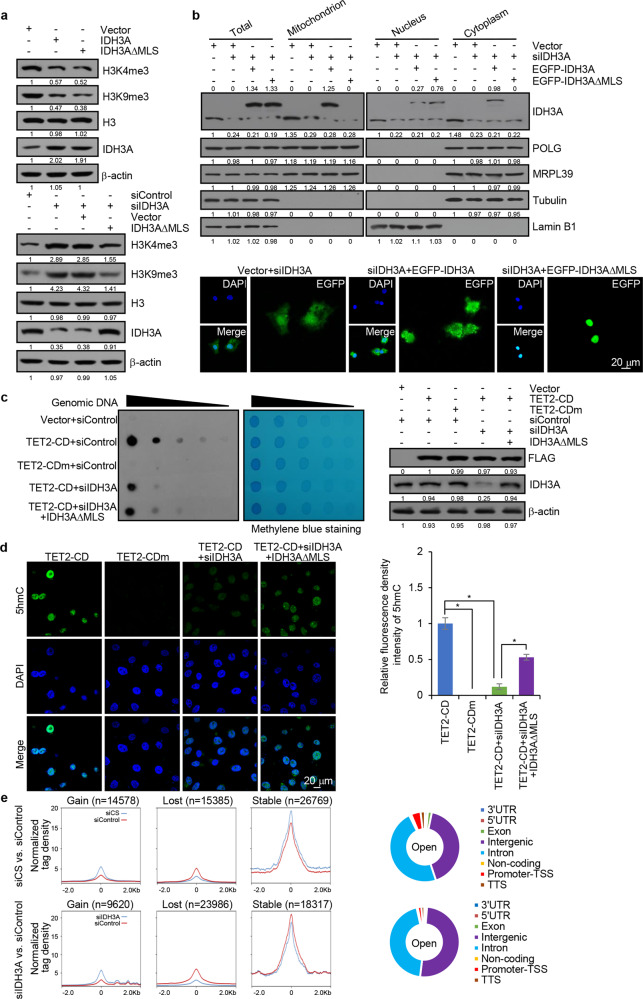
Nuclear IDH3A regulates histone/DNA methylation. **a** HepG2 cells were transfected with IDH3A, IDH3A∆MLS, and/or IDH3A siRNA for western blotting analysis with antibodies against the indicated histone marks or proteins. **b** HepG2 cells treated with IDH3A siRNA were transfected with EGFP-IDH3A or EGFP-IDH3A∆MLS for nuclei/cytoplasm/mitochondria isolation and western blotting analysis of IDH3A expression. The subcellular localization of IDH3A in these cells was analyzed by fluorescence microscopy. Bar, 20 μm. **c** HepG2 cells were co-transfected with FLAG-TET2-CD or FLAG-TET2-CDm and IDH3A siRNA and/or IDH3A∆MLS for dot blotting analysis with anti-5hmC. Spotted DNAs were stained with methylene blue to control the equal loading. The expression of the corresponding proteins was determined by western blotting. **d** HepG2 cells were co-transfected with FLAG-TET2-CD or FLAG-TET2-CDm and IDH3A siRNA and/or IDH3A∆MLS for immunofluorescent staining with anti-5hmC (green). DAPI staining was included to visualize the nucleus (blue). Bar, 20 μm. The relative fluorescent intensity of 5hmC was quantified by Image J software. Error bars represent mean ± SD for triplicate experiments (*p < 0.05). **e** ATAC-seq analysis in CS- or IDH3A-depleted HepG2 cells. The percentage of increased accessible sites in different genic regions is shown. UTR: 5′ and 3′ untranslated regions, TSS: transcription star sites, and TTS: transcription termination sites

IDH3 is one of the rate-limiting enzymes of the TCA cycle, which catalyzes the conversion of isocitrate to α-KG.^26^ It is now clear that a number of histone demethylases as well as the TET dioxygenases require α-KG as a cofactor in their demethylation reactions.^13,14^ To further investigate the functional significance of the nTCA, we next examined the effect of nuclear IDH3A on histone methylation. To this end, IDH3A was overexpressed or knocked down in HepG2 cells, and the effects of IDH3A on histone methylation were analyzed by western blotting of acidly extracted histones. Remarkably, overexpression of either IDH3A or IDH3A∆MLS was associated with a decrease in the level of H3K4me3 and H3K9me3, whereas depletion of endogenous IDH3A was accompanied by an increase in the level of these histone modifications (Fig. 5a). Analogously, reconstitution of nuclear expression of IDH3A with IDH3A∆MLS offset IDH3 depletion-associated increases in histone methylation (Fig. 5a). Subsequently, HepG2 cells treated with IDH3A siRNA were transfected with EGFP-tagged IDH3A or IDH3A∆MLS, and the subcellular localization was determined using western blot and fluorescence microscopy. The results suggested that IDH3A was mainly localized in mitochondrion, whereas IDH3A∆MLS was localized in nucleus, but not in mitochondria (Fig. 5b).

We then sought to determine whether nuclear IDH3A could affect TET activity and DNA cytosine hydroxymethylation. The 5hmC level in most cultured cells is undetectable, but substantially increases in cells transiently expressing the wild-type catalytic domain of TET2 (TET2-CD).^27,28^ Notably, overexpression of TET2-CD, but not the catalytic mutant TET (TET2-CDm),^27,28^ was effective to elevate 5hmC level in HepG2 cells (Fig. 5c), as measured by dot blotting. However, depletion of IDH3A abrogated TET2-CD expression-associated increase in 5hmC level, which could be rescued by expression of IDH3A∆MLS (Fig. 5c). The amount of DNA used in dot blotting was monitored by methylene blue staining (Fig. 5c). In addition, immunofluorescent staining with an antibody against 5hmC in HepG2 cells also showed that ectopic expression of TET2-CD, but not TET2-CDm, resulted in an increase in the fluorescent intensity of 5hmC. However, in IDH3A-deficient cells, overexpression of TET2-CD did not intensify the fluorescent signal of 5hmC, whereas reconstitution of nuclear IDH3A expression by transfection of IDH3A∆MLS in IDH3A-depleted HepG2 cells led to an increased fluorescent intensity of 5hmC (Fig. 5d). Altogether, these data indicate that nuclear IDH3A regulates histone methylation and TET-catalyzed 5hmC production.

Histone modifications and DNA modification represent the integral components of the epigenetic regulatory mechanisms that contribute to the maintenance and modulation of chromatin configurations.^9–11^ To further support the functional connection between the nTCA cycle and epigenetic regulation to extend the above observations to chromatin context, we next investigated the effect of depletion of CS or IDH3A on chromatin dynamics in HepG2 cells using Assay for Transposase-Accessible Chromatin with high-throughput sequencing (ATAC-seq).^29^ Overall, CS deficiency was associated with an increase in the accessibility of chromatin at 14,578 of 56,732 total accessible sites, and IDH3A deficiency was accompanied by an increase in the accessibility of chromatin at 9,620 of 51,923 total accessible sites (Fig. 5e). Detailed analysis indicates that CS and IDH3A mainly influence chromatin accessibility at intergenic, intron, and promoter/transcription start sites (Fig. 5e). These observations support a notion that the functions of nuclear CS and IDH3A are intrinsically linked to chromatin activities.

### The nTCA cycle is functionally linked to transcription regulation and cellular activities

**Fig. 6:**
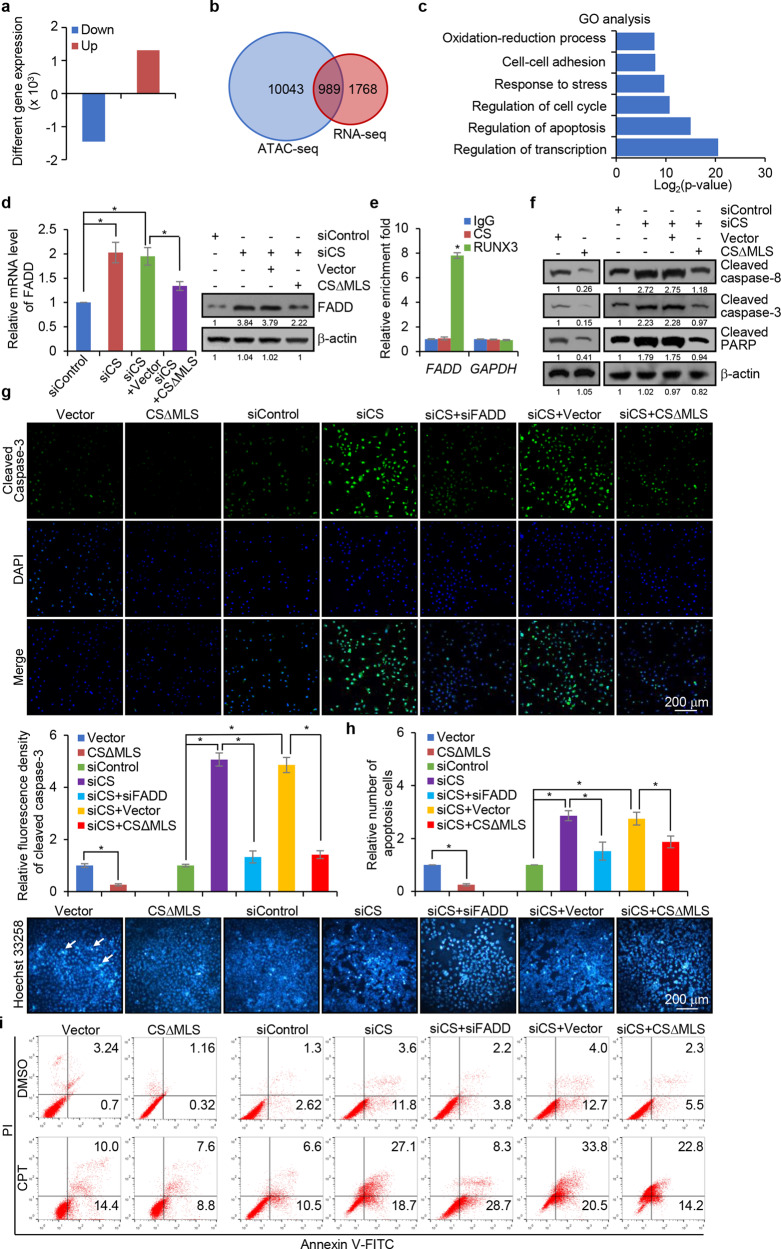
The nTCA cycle is functionally linked to transcription regulation and cellular activities. **a** RNA-seq analysis of differentially expressed genes in CS-depleted HepG2 cells. **b** Venn diagram for cross-analysis of ATAC-seq and RNA-seq in CS-deficient HepG2 cells. **c** Ontology analysis of genes regulated by CS depletion in HepG2 cells. **d** HepG2 cells were transfected with CS siRNA and/or CS∆MLS for analysis of FADD expression by qRT-PCR and western blotting. Error bars represent mean ± SD for triplicate experiments (*p < 0.05). **e** qChIP analysis of *FADD* and *GAPDH* promoter occupancy by CS or RUNX3 in HepG2 cells. Error bars represent mean ± SD for triplicate experiments (*p < 0.05). **f** HepG2 cells were transfected with CS∆MLS or/and CS siRNA followed by treatment with CPT for 48 h for western blotting analysis with antibodies against cleaved caspase-8, caspase-3, and PARP. **g** HepG2 cells were transfected with CS∆MLS or/and CS siRNA or FADD siRNA followed by treatment with CPT for 48 h for immunofluorescent staining with antibodies against cleaved caspase-3 (green). DAPI staining was included to visualize the nucleus (blue). Bar, 200 μm. The relative fluorescent intensity of cleaved caspase-3 was quantified by Image J software. Error bars represent mean ± SD for triplicate experiments (*p < 0.05). **h** HepG2 cells were transfected with CS∆MLS or/and CS siRNA or FADD siRNA followed by treatment with CPT for 48 h for Hoechst 33258 staining and analysis by fluorescence microscopy. The arrows indicate the apoptotic bodies. Bar, 200 μm. **i** HepG2 cells were transfected w**i**th CS∆MLS or/and CS siRNA or FADD siRNA followed by treatment with CPT for 48 h for Annexin V-FITC/PI staining and flow cytometry

To further extend the CS-influenced histone modifications and chromatin accessibility to physiologically relevant responses. CS was depleted in HepG2 cells, and the effect of CS deficiency on the gene expression profile was analyzed by RNA deep sequencing (RNA-seq). The analysis identified 1450 genes that were significantly downregulated and 1307 genes that were significantly upregulated upon CS knockdown (Fig. 6a). Cross-analysis of the RNA-seq data with the data from ATAC-seq correlated 989 genes whose expression was altered upon CS depletion (Fig. 6b). Gene ontology analysis indicated that these 989 genes are implicated in several cellular pathways including apoptosis, cell cycle, and translational initiation (Fig. 6c).

One of the 989 genes is Fas-associating protein with a novel death domain (*FADD*) that is critically involved in the regulation of apoptosis.^30^ To verify that the expression of FADD is modulated by nuclear CS, CS expression was knocked down in HepG2 cells. Measurement by qRT-PCR and western blotting showed that the expression of FADD increased upon CS depletion, an effect that could be offset by reconstituting the expression of nuclear CS through transfecting CS-depleted cells with CS∆MLS (Fig. 6d), indicating that the expression of FADD is modulated by nuclear CS.

To further support this notion and to test the hypothesis that the modulation of FADD expression by CS is derived from the function of the nTCA cycle, not direct binding of CS to the regulatory region of FADD, quantitative chromatin immunoprecipitation (qChIP) assays were performed and possible recruitment of CS on the promoter region of FADD was examined. The results showed that while RUNX3, a known transcriptional regulator of FADD,^31^ was detected on the FADD gene promoter, CS was not detected on this promoter (Fig. 6e).

FADD protein has the potential to form protein aggregates serving as a platform for procaspase-8 recruitment and activation.^30,32,33^ Activated caspase-8 then directly cleaves procaspase-3 or other executioner caspases, eventually leading to apoptosis. To determine whether nuclear CS regulates cell apoptosis through regulation of FADD, we assessed the level of cleaved caspase-8, caspase-3, and PARP in HepG2 cells by western blotting upon CS overexpression or knockdown. We found that overexpression of CS∆MLS led to reduced levels of cleaved caspase-8, caspase-3, and PARP, whereas depletion of CS resulted in increases in the levels of cleaved caspase-8, caspase-3, and PARP, effects that could be reversed by expression of CS∆MLS in CS-depleted HepG2 cells (Fig. 6f). Moreover, immunofluorescent staining also showed that overexpression of CS∆MLS resulted in a decrease in caspase-3 level, whereas depletion of CS led to an increase in caspase-3 level, an effect that was offset by either expression of CS∆MLS or depletion of FADD in CS-depleted HepG2 cells (Fig. 6g). Importantly, Hoechst 33258 staining followed by fluorescent microscopy in HepG2 cells showed that overexpression of CS∆MLS was associated with a decrease in the number of apoptotic bodies, whereas depletion of CS led to an increase in the number of apoptotic bodies, an effect that could be ameliorated by either ectopic expression of CS∆MLS or depletion of FADD (Fig. 6h). Furthermore, flow cytometry analysis in HepG2 cells also revealed that overexpression of CS∆MLS led to a decreased cell apoptosis, whereas CS depletion resulted in an increased cell apoptosis, which could be reversed by either ectopic expression of CS∆MLS or depletion of FADD (Fig. 6i). Altogether, our findings support a notion that nuclear CS is functionally linked to the regulation of gene expression and cellular activity.

**Fig. 7:**
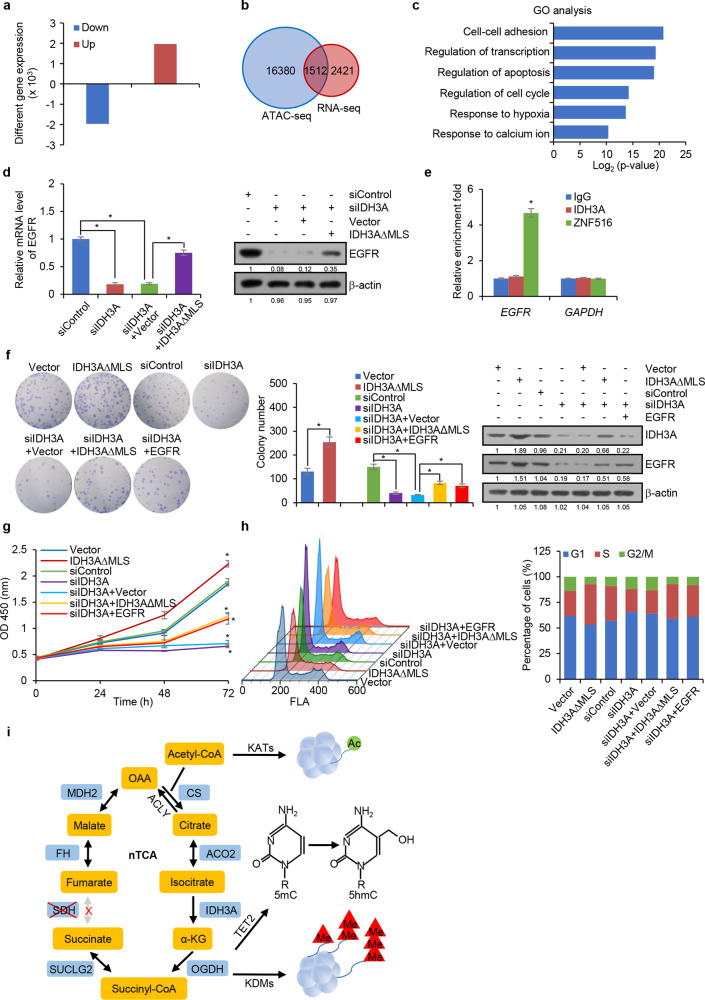
Nuclear IDH3A promotes cell proliferation through regulating EGFR expression. **a** RNA-seq analysis of differentially expressed genes in IDH3A-depleted HepG2 cells. **b** Venn diagram for cross-analysis of ATAC-seq and RNA-seq in IDH3A-depleted HepG2 cells. **c** Ontology analysis of genes regulated by IDH3A depletion in HepG2 cells. **d** HepG2 cells were transfected with IDH3A siRNA and/or IDH3A∆MLS for qRT-PCR and western blotting measurement of EGFR expression. Error bars represent mean ± SD for triplicate experiments (*p < 0.05). **e** qChIP analysis of *EGFR* and *GAPDH* promoter occupancy by IDH3A or ZNF516 in HepG2 cells. Error bars represent mean ± SD for triplicate experiments (*p < 0.05). **f** HepG2 cells were transfected with IDH3A∆MLS or/and IDH3A siRNA or EGFR for colony formation assays. Colonies were stained with crystal violet and counted. Error bars represent mean ± SD for triplicate experiments (*p < 0.05). The expression of IDH3A and EGFR was determined using western blotting analysis. **g** HepG2 cells were transfected with IDH3A∆MLS or/and IDH3A siRNA or EGFR for growth curve measurement and cell proliferation assays using CCK-8 kit. Error bars represent mean ± SD for triplicate experiments (*p < 0.05). **h** HepG2 cells were transfected with IDH3A∆MLS or/and IDH3A siRNA or EGFR for cell cycle analysis by flow cytometry. **i** Proposed model of the nTCA cycle

To extend IDH3A-influenced histone modification and chromatin accessibility to physiologically relevant responses. IDH3A was depleted in HepG2 cells, and the effect of IDH3A deficiency on the gene expression profile was analyzed by RNA-seq. The analysis identified 1,968 genes that were significantly downregulated and 1,965 genes that were significantly upregulated upon IDH3A knockdown (Fig. 7a). Cross-analysis of the RNA-seq data with the data from ATAC-seq correlated 1,512 genes whose expression was altered upon IDH3A depletion (Fig. 7b). Gene ontology analysis indicates that these genes are implicated cellular pathways such as cell cycle, apoptosis, and cellular response to hypoxia (Fig. 7c). One of the 1,512 genes was epidermal growth factor receptor (*EGFR*) that is required for cell proliferation and animal development.^34,35^ To verify that the expression of EGFR is modulated by nuclear IDH3A, IDH3A expression was knocked down in HepG2 cells. Measurement by qRT-PCR and western blotting showed that the expression of EGFR decreased upon IDH3A depletion, an effect that could be rescued by reconstituting the expression of nuclear IDH3A through ectopic expression of IDH3A∆MLS (Fig. 7d).

To further support this notion and to test the hypothesis that the modulation of EGFR expression by IDH3A is derived from the function of the nTCA cycle, not direct binding of IDH3A to the regulatory region of EGFR, qChIP assays were performed and possible recruitment of IDH3A on the promoter region of EGFR was examined. The results showed that while ZNF516, a known transcription regulator of EGFR,^36^ was detected on the EGFR gene promoter, IDH3A was not detected on this promoter (Fig. 7e).

Overexpression or depletion of IDH3A was then performed in HepG2 cells, and the proliferation of these cells was examined by colony formation and CCK-8 assays. The results showed that overexpression of nuclear IDH3A was associated with an increased colony number (Fig. 7f). Consistently, knockdown of IDH3A resulted in a decreased colony number, which could be reversed by ectopic expression of either IDH3A∆MLS or EGFR (Fig. 7f). The expression of IDH3A and EGFR was determined by western blotting (Fig. 7f). Moreover, CCK-8 assays in HepG2 cells showed that overexpression of nuclear IDH3A had a significant promoting effect on cell proliferation, and conversely, depletion of IDH3A overtly inhibited cell proliferation, a phenotype that could be rescued by simultaneous overexpression of either nuclear IDH3A or EGFR (Fig. 7g). Cell-cycle profiling demonstrated that overexpression of nuclear IDH3A was associated with a significant decrease in the percentage of cells in G_1_ phase and an increase in the percentage of cells in S phase (Fig. 7h). Conversely, knockdown of IDH3A resulted in an increase in the percentage of cells in G_1_ phase and a decrease in the percentage of cells in S phase, which could be reversed by ectopic expression of either IDH3A∆MLS or EGFR (Fig. 7h). Altogether, these experiments support a role for nuclear IDH3A in regulating cell proliferation through influencing EGFR expression.

## Discussion

In nearly the last 2 decades, great progress has been made in understanding the inheritable changes in phenotype or gene expression governed by rules other than changes in underlying DNA sequence, the epigenetics. It is now clear that epigenetics not only modulates metabolism but also regulates biological activities ranging from the transfer of information through generations, stem cell differentiation, X chromosome inactivation, tissue regeneration, genomic imprinting, neurological memory processes, to aging.^37,38^ Epigenetics is also important in evolution; the changing environment is currently reshaping the evolution of many species through plastic epigenetic mechanisms.^39^ Epidemiological factors such as diet, environmental changes, microbial infections, and drugs are also influencing our daily lives through epigenetics.^40^ Diseases that have been associated with epigenetic dysregulations range from schizophrenia to cancer.^37,38^ Fortunately, the field of epigenetic therapy is also expanding and novel treatments for numerous diseases derived from epigenetic cues are emerging.

Along with the outstanding development of new technologies that have not only stimulated new discoveries, but also expanded the field, the major driving force in epigenetics has been the efforts to understand the mechanism and significance of the great plasticity of chromatin.^37,38,41^ Even with the study of epigenetics now transcending from planar views to three-dimensional, the essence of epigenetics remains the phenomena related to chemical or structural modifications, leading to the alteration of chromatin configuration.^37,38,41,42^ Post-translational modifications to histones and postreplicational modification of DNA are the hallmarks of epigenetic regulation. These mechanisms act in an orderly manner and coordinated fashion to regulate nuclear activities such as transcription, DNA replication, and DNA repair, in which DNA modifications and histone-modifying activities such as acetylation, phosphorylation, and methylation interplay to form an intimate and self-reinforcing cross-talk and interdependent system to shape the chromatin structure and control the plasticity of chromatin, thereby defining the biological processes.^37,38,41^

It has been long puzzling from the study of epigenetic modifications that a great variety of metabolic intermediates such as acetyl-CoA, α-KG, and succinyl-CoA and cofactors such as FAD and NAD^+^ involved in the mitochondrial TCA cycle are also engaged in epigenetic mechanisms in the nucleus. This issue becomes more complex as the engagement of these metabolites in epigenetic mechanisms pertains to not only the supply, but also the consumption of these molecules. As epigenetic regulations are tightly controlled and highly dynamic and since the TCA cycle and epigenetic regulation are compartmentalized in distinct subcellular locations, a direct fueling of the metabolic intermediates from mitochondria to the nucleus or between mitochondria and the nucleus seems unlikely. Although a few recent studies independently reported the identification of several the mitochondrial TCA cycle-associated enzymes in the nucleus,^16–18^ it seems that only a systematic continuum mechanism of supply/consumption of the Krebs cycle intermediates can meet the tightly controlled and highly dynamic demand of epigenetic regulation. We described in the current study the detection of all the TCA cycle-associated enzymes, except for SDH, in the nucleus. We demonstrated that these nuclear enzymes catalyze biochemical reactions similar to the reactions catalyzed by their counterparts in mitochondria and constitute a nonclassical and incomplete TCA cycle in the nucleus (nTCA cycle). Importantly, we showed that the nTCA cycle is functionally linked to chromatin dynamics and epigenetic regulation. We propose that, similar to the TCA cycle in cyanobacteria, the nTCA cycle could be completed through a series of reverse reactions catalyzed by the enzymes downstream of SDH (Fig. 7i). This system, the nTCA cycle, is a reasonably efficient assembly for supply/consumption of the TCA cycle metabolites in timely and precise epigenetic regulation.

The finding of an additional set of TCA cycle in eukaryotic cells is interesting, and the nTCA cycle is different from the classical TCA cycle in mitochondria is intriguing, yet logic; existence of exactly the same two sets of the TCA cycles in a cell, though in different subcellular compartments, would be rather hard to comprehend. The first question coming with the nTCA cycle is where it is originated. The TCA cycle, like all other cellular systems, is the product of evolution. The origination of the eukaryotes has been considered as one of the major evolutionary innovations in the history of life on this planet. Yet, the emergence of the complex and compartmentalized eukaryotic cells represents a major conundrum in modern biology.^43,44^ Most recent theory considers a symbiogenic scenario in eukaryotic evolution^45^: the emergence of the first eukaryotic cell was triggered by a merger or fusion between an archaeal host cell and an alphaproteobacterial (mitochondrial) endosymbiont.^46–48^ It has been further proposed that the host cell is related to Lokiarchaeota, an archaeal phylum with many eukaryotic characteristics.^49^ As mentioned earlier, cyanobacteria as well as some other prokaryotes also have an incomplete TCA cycle similar to the nTCA cycle.^50^ Thus, it is possible that the nTCA cycle was also originated by symbiogenic archaebacterial inclusion during eukaryote evolution.

The second question coming with the nTCA cycle is how the same TCA cycle-associated enzymes are differentially distributed in mitochondria and the nucleus. One possibility is that the TCA cycle-associated enzymes are labeled by different subcellular localization signaling sequences corresponding to relevant compartments, and nuances in protein sequences could result from alternative splicing, a major transcriptional scheme in eukaryotic cells.^51^ For example, GluN1, the subunit of the N-methyl-D-asparate (NMDA) receptor (NMDAR), has 4 isoforms due to alternative pre-mRNA processing, with GluN1-1a localize in the nucleus whereas other isoforms exist in cytoplasm.^52^ Alternatively, differential post-translational modifications contribute to the differential subcellular compartmentalization of the TCA cycle-associated enzymes in mitochondria and the nucleus; it is well-established that protein post-translational modifications such as phosphorylation, acetylation, and glycosylation play crucial roles in regulating the activity, stability, localization, and interactions or folding of proteins.^53–55^

The third question coming with the nTCA cycle is why and how the nTCA cycle is accommodated in the nucleus. Cyanobacteria lack of the enzyme OGDH,^50^ probably as a consequence of obligate autotrophy.^56^ It has been recognized that the lipoamide-dependent NAD^+^-reducing OGDH typical of most mitochondria, eukaryotes, and aerobic eubacteria is not the only mechanism for decarboxylation of α-KG to produce succinyl-CoA; in various archaeons, such as *Halobacterium*, *Haloferax*, *Archaeoglobus*, and a *Sulfolobus* strain, ferredoxin oxidoreductases could catalyze the reactions associated with eubacterial α-KG and pyruvate dehydrogenases.^57–63^ It has been reported that a *Bradyrhizobium japonicum* mutant lacking OGDH closes the gap in the Krebs cycle by means of a CoA- and NAD(P)^+^- independent oxoglutarate decarboxylase (OGDC), producing succinic semialdehyde.^64^ Recent works proposed that the TCA cycle in cyanobacteria is not “broken” as previously suggested, but uses alternative enzymes, including 2-OGDC and succinic semialdehyde dehydrogenase (SSADH).^65^ Whether it is also a consequence of obligate autotrophy is not clear, the nTCA cycle lacks SDH instead of OGDH. One possibility for this discrepancy could be that the nTCA cycle was originated from cyanobacteria, with an evolutionary shift of missing OGDH to missing SDH. It is even tempting to speculate that, whatever the driving force behind it was, this shift is necessary and advantageous functionally; after all, eukaryotic cells have to distance themselves from archaebacteria in a symbiotic world. Alternatively, the nTCA cycle was originated from an archaebacterium lack of SDH, although such a species is yet to be identified. It is also possible that, for reasons that are currently unknown, only an SDH-lacking TCA cycle is compatible with the evolution of epigenetic deeds in the nucleus. The selective advantage for lack of SDH in the nTCA cycle is currently unclear. Nevertheless, the gaps of the TCA cycle can be closed by a series of reverse reactions, as it is supported by our current study and has been reported for anaerobic microorganisms.^66,67^ ﻿Particularly, ATP-citrate lyase ACLY, which has been found in nuclei can convert citrate into acetyl-CoA ^21^﻿. CS even can catalyze the reaction from citrate to acetyl-CoA in *Hippea maritima* bacteria for autotrophic fixation of carbon with sufficient CO_2_ supply.^68^ In addition, cyanobacteria produce energy phototrophically, rather than by respiration (except under darkness), suggesting that the primary function of their TCA cycle is to produce metabolite precursors for growth.^65^ In fact, many phototropic, lithotrophic, and methylotrophic bacteria utilize oxidative/reductive variants to produce biosynthetic precursors, not energy.^50^ Based on the lack of SDH, which is a heterotetrameric protein complex responsible for oxidation of succinate to fumarate in the TCA cycle and for feeding electrons into the mitochondrial respiratory chain for ATP production (complex II),^69^ we propose that the function of the nTCA cycle is to supply/consume the metabolic intermediates needed/produced in epigenetic regulation, not for energy production, and our experiments support this notion. It is possible that the original nTCA cycle host archaebacteria evolve before the advent of aerobic organisms, and the early anaerobes opted the reactions of the nTCA cycle as a source for biosynthetic precursors, not energy. Importantly, if our interpretation is acceptable, this feature of the nTCA cycle represents an additional line of evidence to support the symbiogenic evolution of eukaryotes.

There is still much to be learnt about the detailed mechanistic and functional differentiation of the nTCA cycle from the classical mitochondrial TCA cycle. For instance, the mitochondrial pyruvate dehydrogenase kinase (PDK) phosphorylates and inhibits mitochondrial PDH, but this kinase was not detected in the nucleus.^70^ The evolutionary background and biochemical significance of lack of SDH in the nTCA cycle remain to be investigated. More importantly, as epigenetic mechanisms as well as other nuclear activities consume a large number of ATPs, the lack of energy production by the nTCA cycle certainly raises questions about the source of nuclear ATPs and the evolutionary consideration of energy usage in eukaryotic cells. Nevertheless, the identification of the existence of the nTCA cycle provides a critical missing link between the metabolism and epigenetics. The functioning of the nTCA cycle fits well to the orderly manner and coordinated fashion of epigenetic mechanisms, to the instant interplays among various epigenetic modifications, and to the timely and precise nature of epigenetic regulation.

## Materials and methods

### Antibodies and reagents

The antibodies used were αCS (SAB2701077), αACO2 (HPA001097), αOGDH (HPA020347), αSUCLG2 (HPA046705), αSDHA (HPA041981), αFH (HPA025770), αMDH2 (HPA019716), αTubulin (T8328), αKAT8 (SAB1306205), αβ-actin (A5441), and αFLAG (F3165) from Sigma; αIDH3A (ab228596), αSDHC (ab191362), αLamin B1 (ab133741), αH3K4me3 (ab8580), αH3K9me3 (ab8898), αH3K9ac (ab4441), αH3K27ac (ab4729), αpan-H3ac (ab47915), αH4K16ac (ab109463), αpan-H4ac (ab240201), αH3 (ab195277), αH4 (ab10158), αcleaved caspase-3 (ab32042), αFADD (ab108601), αKAT2A (ab217876), and αRUNX3 (ab224641) from Abcam; αcleaved caspase-8 (#9496) and αcleaved PARP (#5625) from Cell Signaling Technology; αPOLG (sc-390634) from Santa Cruz; α5hmC (#39069) from Active Motif; αZNF516 (A303-392A) from Bethyl; αMRPL39 (28165-1-AP) from Proteintech. Recombinant human KAT8 protein (SRP5220) was from Sigma and recombinant human KAT2A protein (ab198084) was from Abcam. Protease inhibitor cocktail was from Roche Applied Science. ^13^C_3_-pyruvate, ^13^C_6_-citrate, ^13^C_4_-fumarate and ^13^C_4_-malate were from Cambridge Isotope Laboratories.

### Nuclei isolation

Nuclei PURE Prep Kit (Sigma, NUC-201) was utilized to isolate pure nuclei according to the manufacturer’s instruction. In brief, mouse liver tissues, HepG2 or MCF-7 cells were collected and washed with phosphate-buffered saline (PBS) for three times. Cells were resuspended and lysed with 10 ml ice-cold Nuclei PURE Lysis Buffer (Sigma) containing 0.1% Triton-X-100 and 1 mM DTT on ice for 5 min. After lysis, samples were mixed with 1.8 M sucrose and carefully placed on top of a 1.8 M sucrose gradient. The suspension was then centrifuged at 30,000 × *g* for 50 min. Nuclei at the bottom of the centrifuge were collected and washed with nuclei storage buffer. Purity of nuclei was examined by western blotting and immunofluorescent staining. Isolated nuclei were used immediately for functional experiments. For histone acetylation measurement, HepG2 cells transfected with control siRNA or CS siRNA were maintained in glucose free media 24 h prior to nuclei isolation. Immediately following isolation, nuclei were incubated with 10 mM oxaloacetic acid and 10 mM acetyl-CoA in reaction buffer containing 50 mM Tris-HCl, pH 7.4, 4 mM MgCl_2_, 1 mM NAD^+^ for 4 h at 37 °C. The experiments were terminated by addition of ice-cold reaction buffer and the nuclei were centrifuged at 500 × *g* before they were washed with ice-cold reaction buffer to remove any excess oxaloacetic acid and acetyl-CoA. Nuclei were then prepared for protein extraction and histone acetylation assays by western blotting.

### Cell culture and transfection

HepG2 and MCF-7 cells were obtained from the American Type Culture Collection (ATCC) and maintained in DMEM supplemented with 10% FBS (HyClone), streptomycin (100 mg/mL), and penicillin (100 U/mL) in a humidified incubator equilibrated with 5% CO_2_ at 37 °C. Transfections were carried out using Lipofectamine RNAiMAX Reagent or Lipofectamine 2000 (Invitrogen) according to the manufacturer’s protocol. The sequences of siRNA were: control siRNA, 5′-UUCUCCGAACGUGUCACGU-3′; CS siRNA#1, 5′-GCAUGACGGAGAUGAAUUATT-3′; CS siRNA#2, 5′-GGUAUGAGUUGCCCAUCAUTT-3′; ACO2 siRNA, 5′-UGCUAGAGAAGAACAUUAATT-3′; MDH2 siRNA, 5′-CGACCUGUUCAACACCAAUTT-3′; IDH3A siRNA, 5′-AAGUGUCUACCUGGUAAAUTT-3′; IDH2 siRNA, 5′- AAGAACACCAUACUGAAAGTT-3′; OGDH siRNA, 5′- GGGACAGAUCUGAUUUAUUTT-3′; SUCLG2 siRNA, 5′-AGAAACAGGAAAUUCAAAUTT-3′; SDHA siRNA, 5′-UAAAUCUUCCCAUCUUCAGUU-3′; SDHC siRNA, 5′- AAAUAAGACUCAAAGUUCCCA3′; FH siRNA, 5′-CGGAUAAGUUUGAAAGGAATT-3′; GDH siRNA, 5′-AAAUUUUCUUUCUAAACUCUC-3′; FADD siRNA, 5′-GACCUGGUACAAGAGGUUCTT-3′. Each experiment was performed at least three times.

### Immunofluorescent staining

Cells were grown on coverslips and fixed in 4% paraformaldehyde, permeabilized with 0.2% Triton-X-100 in PBS, blocked with 0.4% BSA, and incubated with appropriate primary antibody at 4 °C overnight. The slides were then washed with PBS containing 0.1% Triton-X-100 followed by incubation with fluorescently labeled secondary antibody at room temperature for 1 h. Meanwhile, 0.1 μg/ml DAPI (Sigma) was added to stain nuclei and 100 nM Mitotracker Red (Cell Signaling Technology) was used to stain mitochondrion. To analyze the level of activated caspase-3, transfected HepG2 cells were treated with 1 μM camptothecin (CPT, Selleck) for 48 h prior to staining with anti-cleaved caspase-3 (Cell Signaling Technology). The images were visualized using an Olympus or Zeiss confocal microscope. The relative fluorescence intensity was analyzed by Image J software. Each experiment was performed at least three times.

### Metabolite extraction and analysis

Isolated nuclei were suspended in 500 μl of ice-cold 80% methanol and 20% ddH_2_O. The samples were then placed in −80 °C for 30 min and centrifuged at 13,000 × *g* for 20 min to pellet proteins, lipids, and cell debris. The supernatants were evaporated in a vacuum drier (SpeedVac), dried sample was dissolved in HPLC-grade H_2_O to for analysis by LC-MS. The metabolite level was normalized to protein concentration. Each experiment was performed at least three times.

### Enzymatic activity assay

The activity of the enzymes related to the TCA was determined based on the colorimetry using commercial kits (BioVision), including CS Activity Colorimetric Assay Kit (K318), IDH Activity Colorimetric Assay Kit (K756), OGDH Activity Colorimetric Assay Kit (K678), SCS Activity Colorimetric Assay Kit (K597), SDH Activity Colorimetric Assay Kit (K660), FH Activity Colorimetric Assay Kit (K596), MDH Activity Colorimetric Assay Kit (K654) and GDH Activity Assay Kit (K729). The positive control samples were provided from the kit, and the preparation methods were as follows: the SCS, SDH, or OGDH in positive control tube was reconstituted with 100 µl corresponding assay buffer, FH with 200 μl dH_2_O, MDH with 400 µl assay buffer, GDH with 220 µl assay buffer, CS with 1000 µl assay buffer; 5 μl above solution was used for the reaction as positive control, respectively. For metabolic flux analysis (MFA), isolated nuclei from HepG2 cells was incubated with 10 mM ^13^C_3_-pyruvate, ^13^C_6_-citrate, ^13^C_4_-malate, or ^13^C_4_-fumarate and acetyl-CoA at 37 °C for 4 h in a 200 μl reaction mixture containing 50 mM Tris-HCl, pH 7.4, 4 mM MgCl_2_, 1 mM NAD^+^. At the end of the reaction, the isotope-labeled metabolite was extracted and detected by LC-MS. Each experiment was repeated biologically at least three times.

### Michaelis constant (*K*_M_) of nuclear CS measurement

For detection of *K*_*M*_ of nuclear CS, the nucleus of HepG2 cells transfected with His-CS was isolated using Nuclei PURE Prep Kit (Sigma), and lysed with lysis buffer (1% NP-40, 20 mM sodium phosphate, 500 mM NaCl, 50 mM imidazole, pH 7.4) containing protease inhibitor cocktail (Roche) and phosphatase inhibitor (Applygen). Nuclear extracts were applied to the IDA-Ni magnetic agarose beads (BEAVER Catalog: 70501-K10) to adsorb His-CS at 4 °C for 1 h. After binding, the beads were washed with wash buffer (20 mM sodium phosphate, 500 mM NaCl, 100 mM imidazole, pH 7.4) at three times, and then His-CS was eluted with elution buffer (20 mM sodium phosphate, 500 mM NaCl, 500 mM imidazole, pH 7.4). Ten microliters of His-CS eluent was incubated with 10 mM oxaloacetic acid and 0–40 mM acetyl-CoA at 37 °C for 30 min in a 200-μl reaction mixture containing 50 mM Tris-HCl, pH 7.4, 4 mM MgCl_2_, and 1 mM NAD^+^. The production of citrate was measured using Citrate Colorimetric/Fluorometric Assay Kit (BioVison, K655) according to the manufacturer’s protocol. The *K*_*M*_ of nuclear CS was analyzed by double-reciprocal transformation of Lineweaver and Burk.

### ATP content measurement

Mitochondria from HepG2 cells were obtained using the Mitochondrial Extraction Kit from Solarbio according to the manufacturer’s protocol. Isolated mitochondria and nuclei from HepG2 cells were incubated with 10 mM oxaloacetic acid and 10 mM acetyl-CoA at 37 °C for 30 min in a 200-μl reaction mixture containing 50 mM Tris-HCl, pH 7.4, 4 mM MgCl_2_, and 1 mM NAD^+^. The ATP content was measured by ATP Colorimetric/Fluorometric Assay Kit (Beyotime Biotechnology) according to the manufacturer’s instruction. Each experiment was repeated biologically at least three times.

### Western blotting

Cells were lysed using lysis buffer (50 mM Tris-HCl, pH 7.5, 2 mM EDTA, 0.5% NP-40 and 150 mM NaCl) with the protease inhibitor cocktail from Roche for 30 min at 4 °C, followed by centrifugation at 14,000 × *g* for 15 min at 4°C. The BCA Protein Assay Kit (Thermo Fisher Scientific) was used to measure the protein concentration. Equal amounts of lysates (40 μg) were resolved on 10% sodium dodecyl sulphate–polyacrylamide gel electrophoresis (SDS-PAGE) gels and transferred onto nitrocellulose membranes. After blocked with 5% fat-free milk in TBS-Tween 20 for 1 h at room temperature, the membranes were incubated with appropriate antibodies at 4 °C overnight, followed by incubating with secondary antibodies for 1 h at room temperature. Western blotting luminol reagent (Santa Cruz) was used to visualize the immunoreactive bands according to the manufacturer’s protocol. Each experiment was performed at least three times. The densitometric ratio for proteins was measured by Image J software (National Institutes of Health, version 1.53e).

### Histone deacetylation and acetyltransferase assay

For deacetylation assay, 10 μg calf thymus bulk histones (Sigma) were incubated with 0.25–5 μg of GST-CS or GST-HDAC3 in deacetylation assay buffer (10 mM Tris, pH 8.0, 150 mM NaCl, 1 mM MgCl_2_) at a final volume of 15 μl for 2 h at 37 °C. The reaction mixture was boiled in SDS buffer, resolved on SDS-PAGE, and subjected to mass spectrometry. For acetyltransferase assay, recombinant human KAT2A or KAT8 was incubated with 10 μg calf thymus histones and 10 mM acetyl-CoA in the presence or absence of 10 mM oxaloacetate with or without addition of affinity-purified CS from HepG2 nuclei in a 15 μl reaction mixture containing 50 mM Tris-HCl, pH 8.0, 5% glycerol, 0.1 mM EDTA, 1 mM dithiothreitol, 5 mM PMSF at 30 °C for 30 min. The reaction materials were then resolved on 15% SDS-PAGE and transferred onto nitrocellulose membranes to detect histone acetylation. Each experiment was performed at least three times.

### RT-PCR and real-time RT-PCR (qRT-PCR)

Total cellular RNAs were isolated from samples with the Trizol reagent (Invitrogen). First strand cDNA was synthesized using the Reverse Transcription System (TransGen Biotech). Quantitation of all gene transcripts was done by qRT-PCR using Power SYBR Green PCR Master Mix and an ABI PRISM 7500 sequence detection system (Applied Biosystems) with the expression of GAPDH as the internal control. The primer pairs used were: FADD forward: 5′-GTGGCTGACCTGGTACAAGAG-3′ and reverse: 5′-GGTAGATGCGTCTGAGTTCCAT-3′; EGFR forward: 5′-AGGCACGAGTAACAAGCTCAC-3′ and reverse: 5′-ATGAGGACATAACCAGCCACC-3′; GAPDH forward: 5′-CCCACTCCTCCACCTTTGAC-3′ and reverse: 5′-CATACCAGGAAATGAGCTTGACAA-3′. Each experiment was performed at least three times.

### RNA-seq

For RNA-seq experiments, HepG2 cells were treated with control siRNA, CS siRNA, or IDH3A siRNA prior to RNA extraction. Three biological replicates were processed for each group. The cDNA synthesis and library construction were conducted as described previously.^71^ Libraries were sequenced using HiSeq 2500 (Illumina) with 2 × 150 bp pair-end sequencing by the Centre for Applied Genomics (SickKids). Qualified data were mapped to hg19 genome using STAR.^72^ Gene expression level was analyzed and annotated with Cuffdiff software. Genes defined as differentially expressed were selected with FPKM > 1, and *p*-value < 0.05. R-studio software was used to visualize the analyzed results. Cluster Profiler package in R was used for Gene Ontology (GO) analysis with the cutoff of *q*-value < 0.05.

### ATAC-seq

Nuclei were isolated from 50,000 cells and subjected to transposase reaction at 37^ o^C for 30 min. Libraries were generated as described previously.^29^ Sequence data were aligned to hg19 genome by Bowite2. After sorting with Samtools, peaks were called on the set of all ex vivo ATAC-seq reads using MACS2 and *q*-value < 0.05 as a cutoff value.^73^ Peaks annotation was conducted by Homer. Promoter region defined as ±1 kb from TSS. Differential accessibility was assessed using Bedtools and Homer as well. Other analysis was performed using deeptools.^74^ Cluster Profiler package in R were used for analyzing GO with the cutoff of *q*-value < 0.05. Each experiment was repeated twice biologically.

### ChIP and qChIP

ChIP assays were performed essentially the same as previously described.^75^ The enrichment of DNA templates was analyzed by conventional PCR using primers: *FADD* forward: 5′-TGCATTTTCCACGTTTGTCCC-3′ and reverse: 5′-CTGCCGAGTCCACCTGTTTG-3′; *EGFR* forward: 5′-GTAGAGCCCGTTCCGTTGTC-3′ and reverse: 5′-AAGCACCACCCATGTGCTTTA-3′; *GAPDH* forward: 5′-TCCTCCTGTTTCATCCAAGC-3′ and reverse: 5′-TAGTAGCCGGGCCCTACTTT-3′. Each experiment was performed at least three times.

### Dot blotting

Dot blotting was performed as described previously with minor modifications.^76^ In brief, HepG2 cells were co-transfected with FLAG-TET2-CD or TET2-CDm and IDH3A siRNA and/or IDH3A∆MLS for 48 h. DNA was isolated and spotted on a nitrocellulose membrane followed by crosslinking with an ultraviolet lamp for 15 min. The membrane was then blocked with 5% fat-free milk for 1 h at room temperature and incubated with anti-5hmC (Active Motif) at 4 °C overnight. After incubation with HRP-conjugated secondary antibody for 1 h at room temperature, the membrane was washed with for three times and visualized by western blotting luminol reagent (Santa Cruz). The membrane was also stained with methylene blue to control equal loading. Each experiment was performed at least three times.

### Hoechst 33258 staining

HepG2 cells were transfected with CS∆MLS or/and CS siRNA or FADD siRNA and treated with 1 μM CPT for 48 h followed by Hoechst 33258 staining for 10 min in dark and washing with PBS for three times. The morphology of the nuclei was examined by fluorescence microscopy (magnification, ×10). Each experiment was performed at least three times.

### Apoptosis analysis

Annexin V-FITC Apoptosis Detection Kit (Vazyme) was used for apoptosis analysis according to the manufacturers’ instruction. Briefly, HepG2 cells were transfected with CS∆MLS or/and CS siRNA or FADD siRNA and treated with DMSO or 1 μM CPT for 48 h. The cells were trypsinized, washed with PBS, resuspended in Annexin V binding buffer and then stained with Annexin V-FITC and propidium iodide (PI) for 15 min in dark at room temperature. A minimum of 1 × 10^4^ cells per sample were acquired and analyzed using the FACSCalibur flow cytometer (BD Biosciences). Each experiment was performed at least three times.

### Colony formation assay

ChIP assays were performed essentially the same as previously described.^77^ HepG2 cells were transfected with IDH3A∆MLS or/and IDH3A siRNA or EGFR. About 1 × 10^3^ cells were then seeded onto 6-well culture plates. After incubation for 14 days, cells were washed for three times with PBS and fixed with 4% paraformaldehyde. The colonies were stained with 0.5% crystal violet solution and counted using a light microscope. Each experiment was performed at least three times.

### Cell proliferation analysis

HepG2 cells were transfected with IDH3A∆MLS or/and IDH3A siRNA or EGFR. About 2000 transfected cells were seeded onto 96-well plates with 200 μl of DMEM. Every 24 h, the cells were treated with 20 μl of Cell Counting Kit-8 (CCK-8 Kit, Beyotime Biotechnology) solution and incubated for another 90 min. The absorbance of each well was measured at 450 nm using VARIOSKAN FLASH (Thermo Fisher Scientific). The blank control containing 20 μl of CCK-8 solution in 200 μl of DMEM was measured and subtracted, and the cell proliferation curve was drawn. Each experiment was performed at least three times.

### Flow cytometry

HepG2 cells were transfected with IDH3A∆MLS or/and IDH3A siRNA or EGFR for 48 h and collected. The cells were washed with PBS for three times and fixed in 70% ethanol at −20 °C overnight. After being washed with PBS, cells were incubated with 200 μg/ml RNase A (Sigma) in PBS for 30 min at 37 °C and then stained with 50 μg/ml propidium iodide (PI) for 10 min. Cell cycle was measured by FACS Calibur (Becton Dickinson) and analyzed with ModFit LT 3.0 (Verity Software House Inc). Each experiment was performed at least three times.

### Statistical analysis

Results are presented as mean ± SD unless otherwise noted. SPSS version 18.0 was used for statistical analysis. Student’s *t*-test was conducted to determine the significance. One-way analysis of variance (ANOVA) with Tukey’s post hoc analysis was performed to compared multiple groups.

## Supplementary information

Supplemental figure

## Data Availability

All data that support the findings of this study are available from the corresponding author upon reasonable request.
