# Supplementary material for: The existence of a nonclassical TCA cycle in the nucleus that wires the metabolic-epigenetic circuitry
Source: Signal Transduct Target Ther. 2021 Nov 3;6:375. doi: 10.1038/s41392-021-00774-2 (PMC8563883; doi:10.1038/s41392-021-00774-2)
Supplement: Supplementary file 1 — Supplemental figure [file 41392_2021_774_MOESM1_ESM.docx]

Supplementary Materials for

**The Existence of a Nonclassical TCA Cycle in the Nucleus that Wires the Metabolic-Epigenetic Circuitry**

Xujun Liu^1,2,6^, Wenzhe Si^1,6^, Lin He^1^, Jianguo Yang^1^, Yani Peng^1^, Jie Ren^1^, Xiaoping Liu^1^, Tong Jin^1^, Huajing Yu^1^, Zihan Zhang^1^, Xiao Cheng^1^, Wenting Zhang^1^, Lu Xia^1^, Yunchao Huang^1^, Yue Wang^3,4^, Shumeng Liu^4^, Lin Shan^4^, Yu Zhang^1^, Xiaohan Yang^1,5^, Haixia Li^2^, Jing Liang^1^, Luyang Sun^1,5,7^, and Yongfeng Shang^1,3,4,7^

Correspondence to: Luyang Sun (luyang_sun@hsc.pku.edu.cn), Yongfeng Shang (yshang@hsc.pku.edu.cn)

**This PDF file includes:**

Figure. S1 to S4


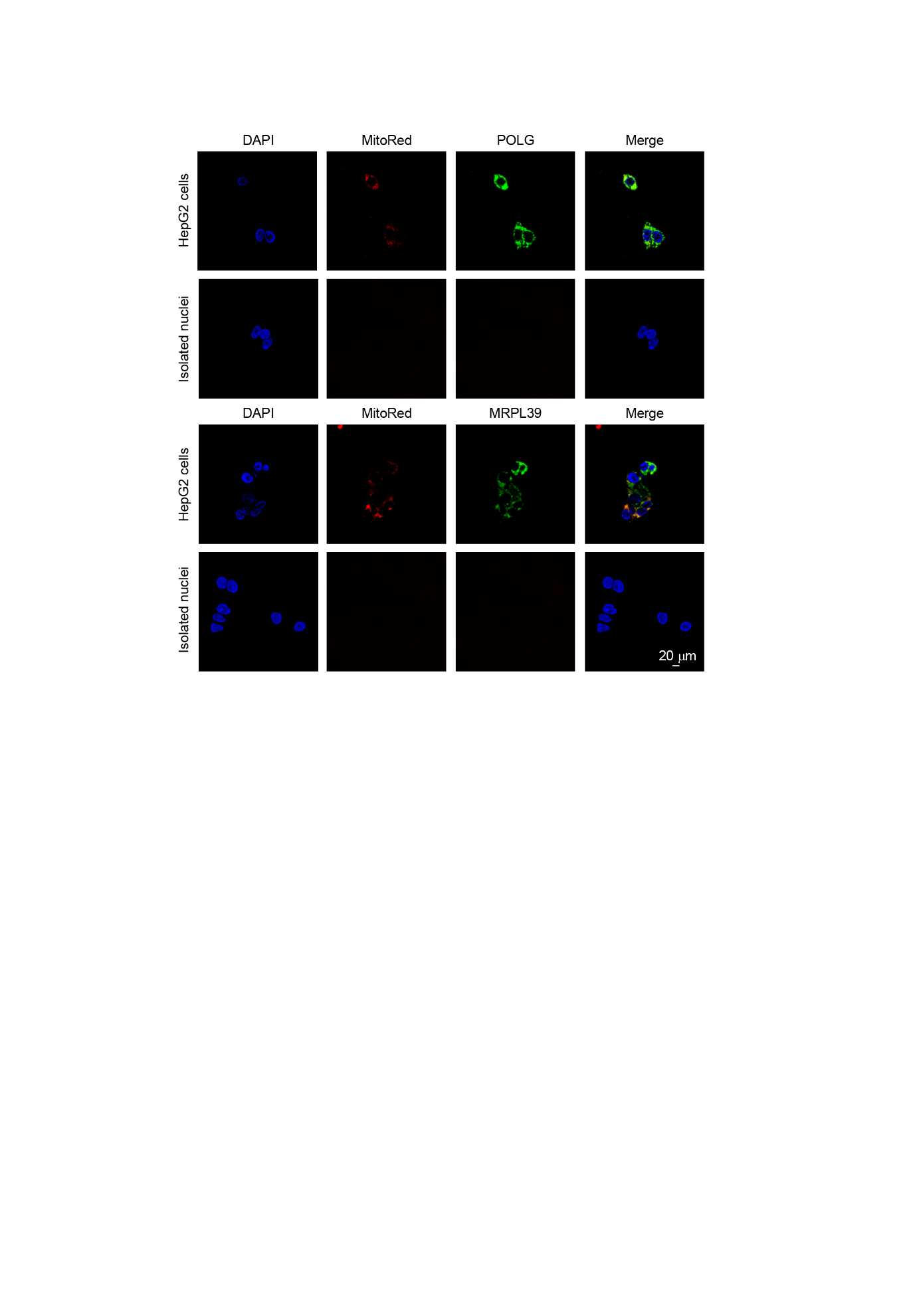
Figure. S1.

Nuclei were isolated from HepG2 cells using a nuclei-specific, high sucrose gradient centrifugation. The HepG2 cells and isolated nuclei were stained with MitoTracker Red (red), DAPI (blue), and POLG or MRPL39 (green). Bar, 20 μm.


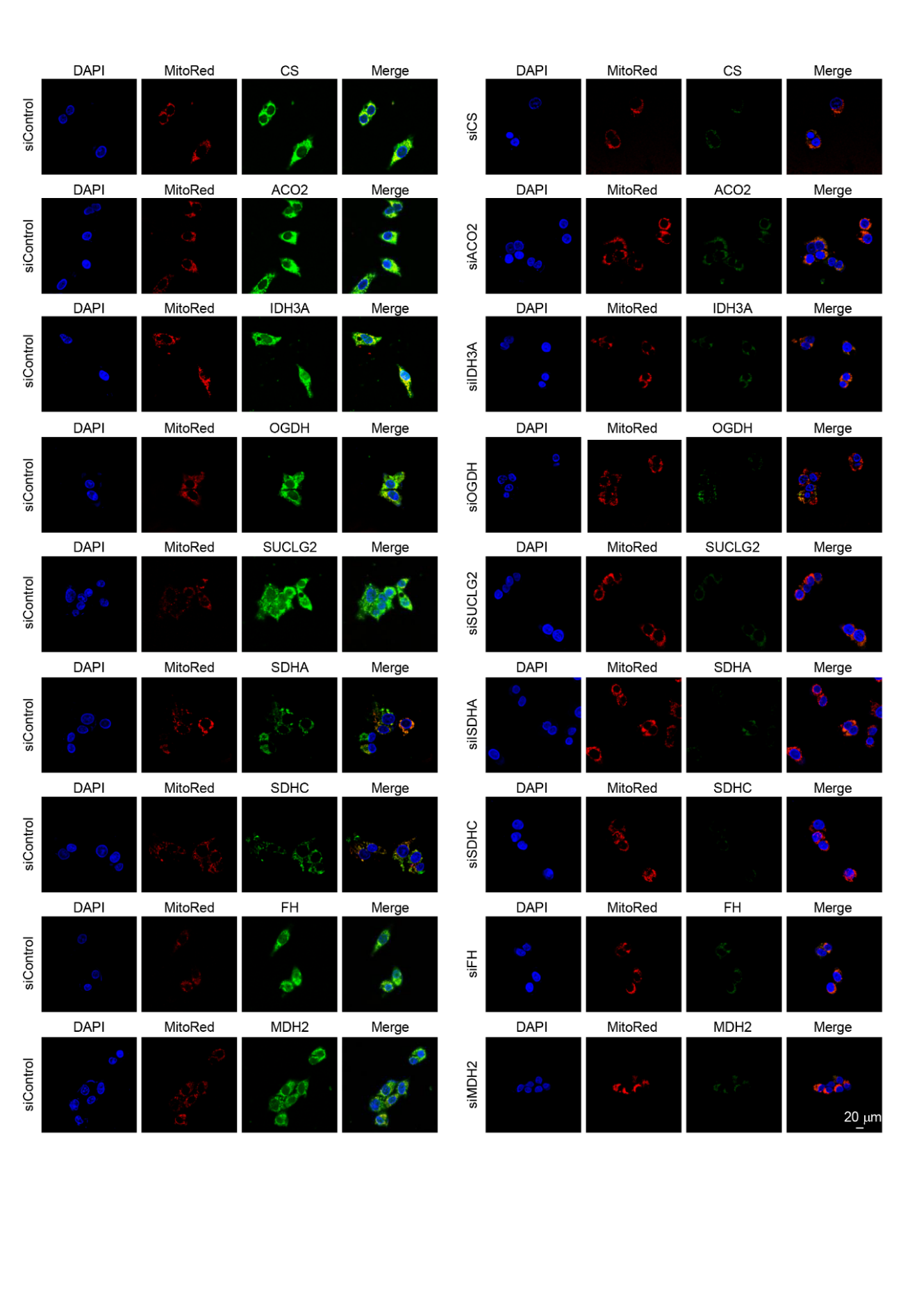
Figure. S2.

HepG2 cells were transfected with control siRNA or indicated siRNAs, and then immunostained with antibodies against the TCA cycle-associated enzymes (green) or MitoTracker Red (red). DAPI staining was used to visualize the nucleus (blue). Bar, 20 μm.

Figure. S3.


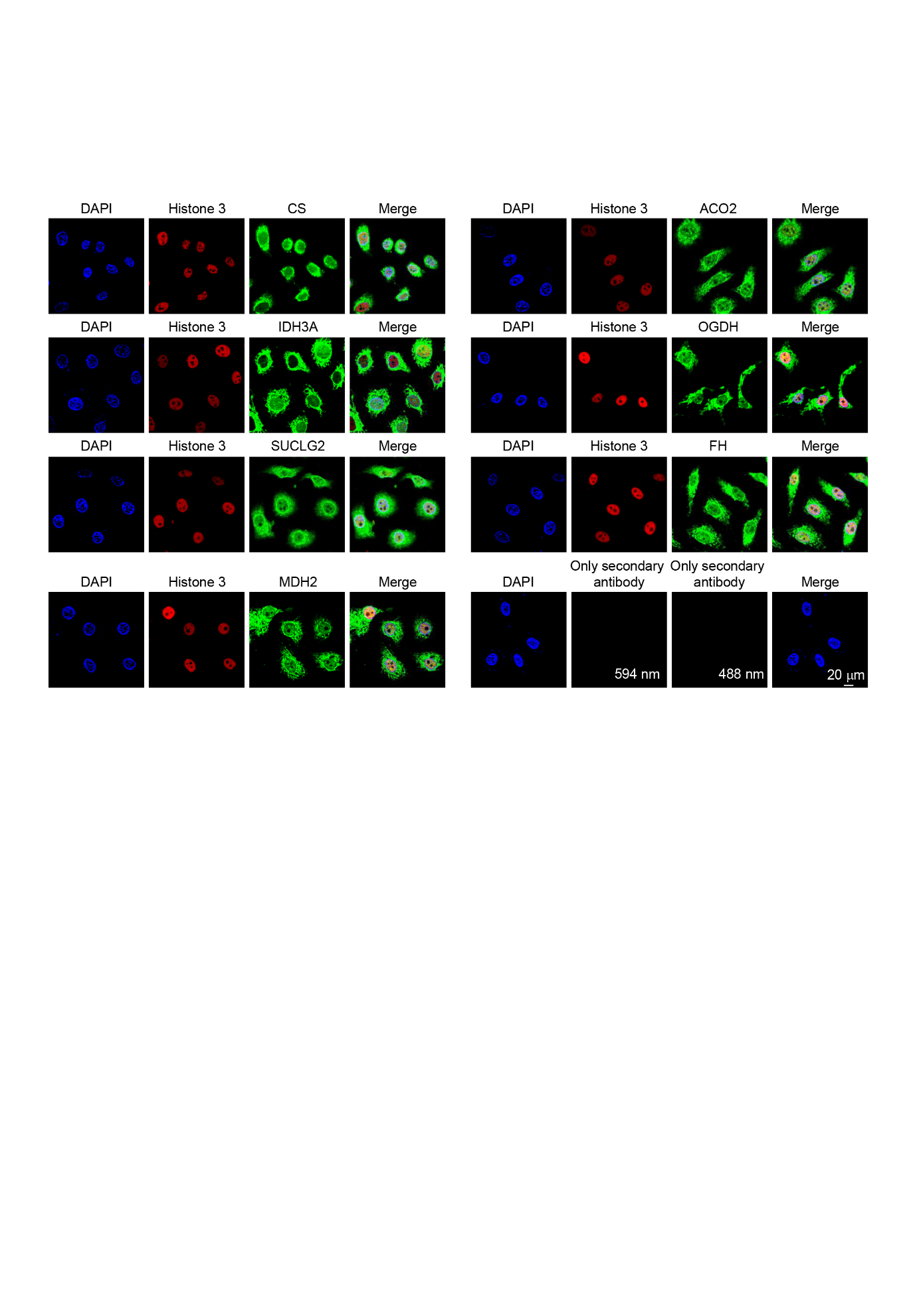
HepG2 cells were immunostained with antibodies against the TCA cycle-associated enzymes or histone H3 (red). Secondary-only antibody to Alexa Fluor^®^ 488 and 594 was included as negative controls to validate the specificity of the antibodies used. DAPI staining was included to visualize the nucleus (blue), and MitoTracker Red was used to stain mitochondria (red). Bar, 20 μm.


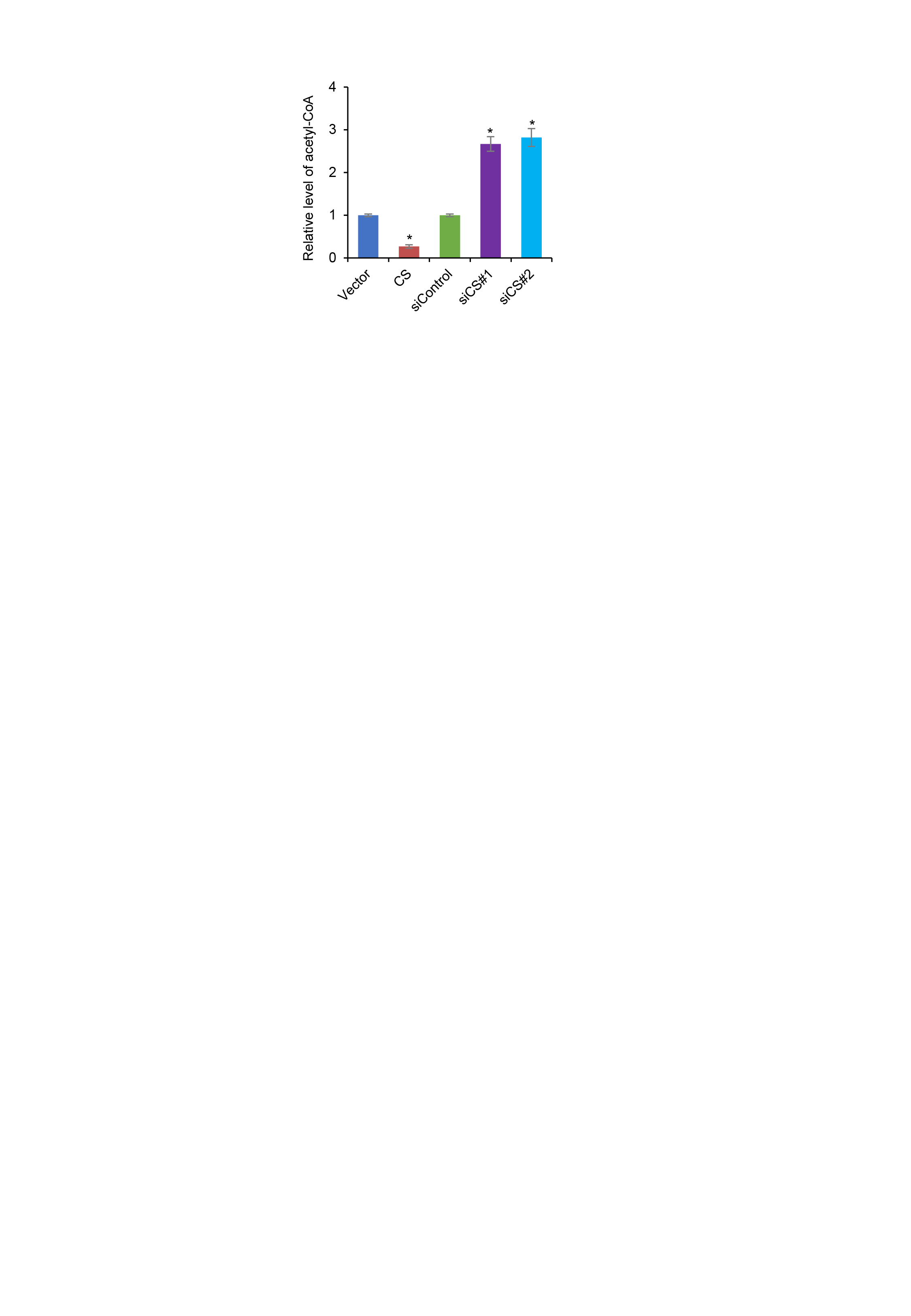
Figure. S4.

The nuclei of HepG2 cells transfected with FLAG-CS or treated with CS siRNA were isolated, and the level of acetyl-CoA within the nuclear compartment was detected using Acetyl-CoA Activity Assay Kit. Error bars represent mean ± SD for triplicate experiments (*p < 0.05).
